# Are lizards sensitive to anomalous seasonal temperatures? Long-term thermobiological variability in a subtropical species

**DOI:** 10.1371/journal.pone.0226399

**Published:** 2019-12-19

**Authors:** André Vicente Liz, Vinicius Santos, Talita Ribeiro, Murilo Guimarães, Laura Verrastro

**Affiliations:** 1 Programa de Pós–Graduação em Biologia Animal, Departamento de Zoologia, Instituto de Biociências, Universidade Federal do Rio Grande do Sul, Porto Alegre, Rio Grande do Sul, Brazil; 2 Programa de Pós–Graduação em Ecologia, Departamento de Ecologia, Instituto de Biociências, Universidade Federal do Rio Grande do Sul, Porto Alegre, Rio Grande do Sul, Brazil; Wildlife Conservation Society Canada, CANADA

## Abstract

Alterations in thermal niches have been widely associated with the Anthropocene erosion of reptiles’ diversity. They entail potential physiological constraints for organisms’ performance, which can lead to activity restrictions and impact fitness and demography. Reptiles are ectotherms which rely on seasonal periodicity to maximize the performance of biological functions. Despite it, the ecological implications of shifts in local temperatures are barely explored at the seasonal scale. This study aims to assess how changes in air temperature and substrate temperature affect the activity, body temperature (*T*_b_) and thermoregulation patterns of the sand lizard, *Liolaemus arambarensis* (an endangered, microendemic species from southern Brazil), throughout a four-year period. Field surveys were conducted monthly on a restricted population in a sand-dune habitat. The annual fluctuations of the seasonal temperatures led to significant changes in the activity and *T*_b_ of *L*. *arambarensis* and shaped thermoregulation trends, suggesting biological plasticity as a key factor in the face of such variability. Lizards tended to maintain seasonal *T*_b_ in mild and harsh seasons through increased warming/cooling efforts. Anomalous winter conditions seemed especially critical for individual performance due to their apparent high impact favouring/constraining activity. Activity and thermoregulation were inhibited in frigid winters, probably due to a vulnerable physiology to intense cold spells determined by higher preferred body temperatures than *T*_b_. Our results warn of a complex sensitivity in lizards to anomalous seasonal temperatures, which are potentially enhanced by climate change. The current work highlights the importance of multiannual biomonitoring to disentangle long-term responses in the thermal biology of reptiles and, thereby, to integrate conservation needs in the scope of global change.

## Introduction

For almost two decades, we have been aware that reptiles are globally declining and that climate change is a major driving factor [1,2]. Reptiles are greatly constrained by external temperatures due to their need to thermoregulate [3,4], which makes them very susceptible to environmental changes and anthropogenic impacts [5,6]. Their physiology (e.g. metabolic rate), phenology (e.g. reproduction onset, voltinism), ecological relations and other life-history traits (e.g. growth, sexual maturity, longevity) are sensitive to thermal shifts, being important factors associated with populations’ persistence [7–11]. In this respect, various studies have forecasted multiple population and species extinctions within the century, due to alterations in local thermal niches [8,12–14]. Understanding reptiles resilience and plasticity towards thermal changes has become, therefore, imperative for integrative conservation [15,16].

Global warming is possibly the most blatant phenomenon stemming from the climatic alterations in the Anthropocene. Large-scale predictions on demographic declines (e.g. [12,14,17]) and models of climate change buffering (e.g. [18,19]) generally agree about the response of reptiles to increasing temperatures. However, while the impact of global warming on biodiversity is widely acknowledged, other effects of climate change may be overlooked (e.g. extreme weather spells or seasonal climate distortions; [16,20,21]). Alterations in seasonal temperatures are notable phenomena [22–25], though their biological implications remain unexplored.

In order to optimise their biological activities [3,4], reptiles correlate their activity and body temperature (*T*_b_) patterns with seasonal dynamics of environmental temperatures [26–28], which are often closely related to their phenological traits. Temperatures approaching organisms’ tolerance thresholds may impose serious challenges to their thermoregulation capacities and prompt physiological instability, leading to activity limitations [3,4]. Reductions in the available foraging or mating time have severe ecological implications and negatively impact fitness, demography and distribution range [14,29–31]. Biological consequences may be especially severe at certain stages of the organism´s annual cycle, such as the reproductive season or in gonad growth and gametogenesis peaks, when foraging limitation becomes a major threat due to increasing energy demands [29,32,33].

Lizards are among the most sensitive reptiles to climate change [2,14,17,34–37]. As behavioural thermoregulators, they adjust their *T*_b_ by interacting with external heat sources and by performing combinations of specific actions, such as adjustment of activity periods, body positions and postures, as well as their microhabitat use [7,38,39]. Species whose *T*_b_ balance mostly depends on substrate temperatures are thigmothermic, while those primarily relying on solar irradiation are heliothermic. Moreover, species range from marked thermoregulators to thermoconformers and from thermal generalists to thermal specialists, according to their thermoregulatory capabilities and thermal sensitivities. Such traits characterize the thermal biology of a species, which is a combination of historical factors (i.e. phylogeny) and adaptations to particular environments [3]. Thermobiological features follow a complex, continuum range and may vary along with external conditions, allowing individuals to minimize associated costs [39,40].

Microendemic lizards are particularly exposed to thermal alterations because they restrict their thermal preferences to the local niches available within a limited space, and are often unable to colonize new suitable habitats [18, 34,41]. Tropical and subtropical species living at low altitudes are also highly vulnerable, due to the presumed strong impact of global warming on these latitudes [14,25,35]. Despite this, the thermal biology of neotropical lizards has been rarely assessed in relation to trends in climate variability. *Liolaemus* spp. (Wiegmann, 1883) represent one of the most remarkable adaptive radiations among Squamata reptiles. These organisms colonized a wide array of South American habitats and combine a largely diverse taxonomy (>250 species), ecology, morphology and life-story traits, which makes them excellent models for addressing evolutionary and ecological questions [42]. Yet, while *Liolaemus* thermal biology has been targeted in species from cold, extreme environments in the Andes and Patagonia [43–45], other regions remain understudied. Besides, the lack of long-term assessments constrains unlocking potential uncharted aspects of its adaptive evolution, despite the lability of *Liolaemus T*_b_ in comparison to preferred body temperatures (*T*_pref_).

Biomonitoring constitutes a useful, although uncommon, mechanism to track changes in animal-environment interactions across different time scales [46]. This study presents a multiannual, year-round approach to the thermal biology of the subtropical, microendemic sand lizard, *L*. *arambarensis*. Specifically, we assess potential changes in the influence of air temperature (*T*_a_) and substrate temperature (*T*_s_) on species activity and *T*_b_ patterns throughout the annual cycle, between years and in specific seasons over different years. We tested the following hypotheses: (1) lizard activity and *T*_b_ patterns align with the dynamics of environmental temperatures, defined here as air and surface temperatures, across the annual cycle; (2) the relative influence of *T*_a_ and *T*_s_ on lizard’s *T*_b_ balance changes in cold and warm seasons; (3) lizards present higher activity in thermally mild seasons compared to thermally harsh seasons (i.e. year-to-year comparisons of the same season); (4) lizards present similar *T*_b_ and different levels of active thermoregulation between mild and severe seasons.

## Material and methods

### Study species and site

**Fig 1:**
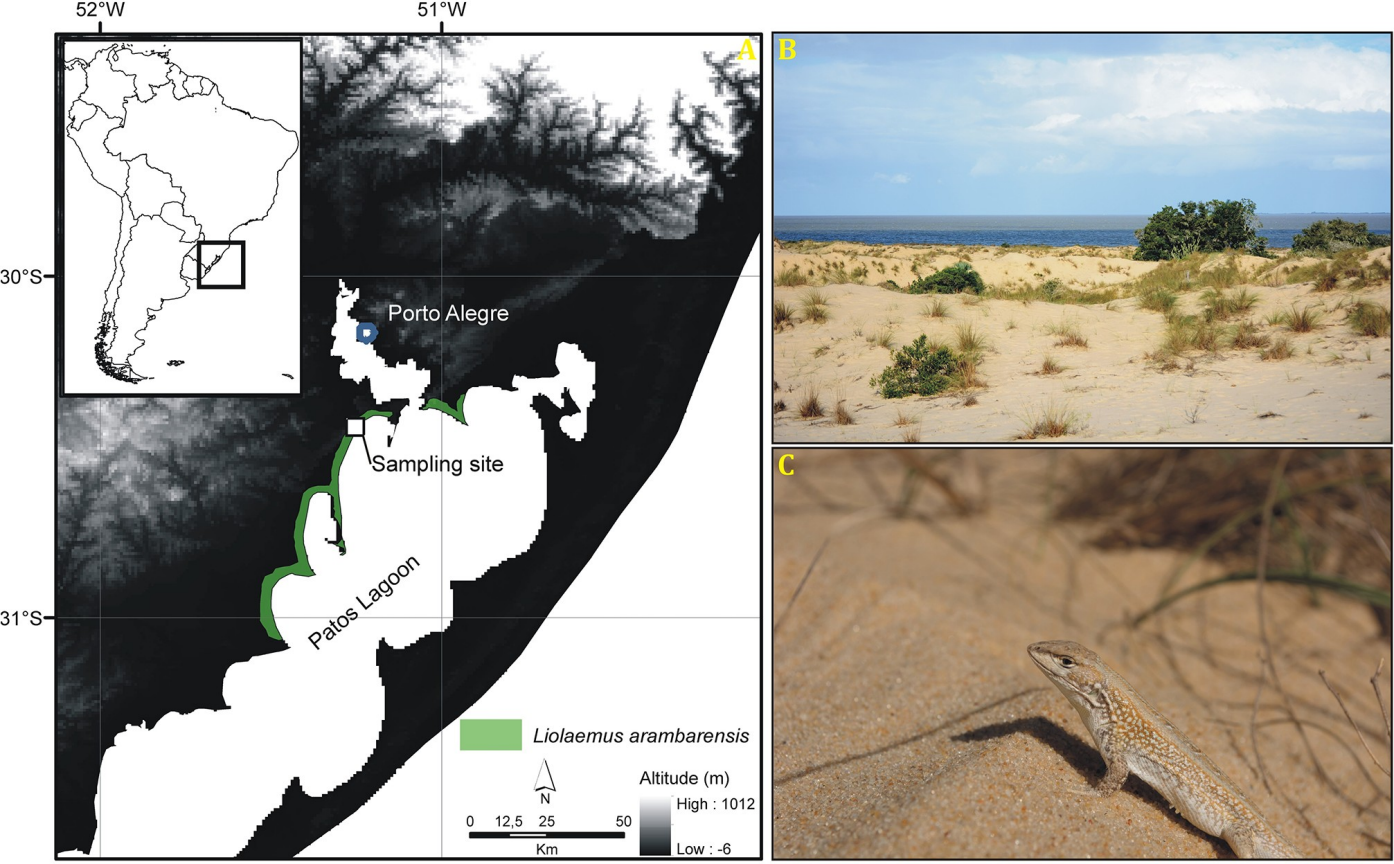
Study area and species. (A) The dark-grey square corresponds to the sampling site, in the RPPN Barba Negra, Barra do Ribeiro, RS, Brazil. The white-grey striped area represents the distribution of *Liolaemus arambarensis*. (B) Image of the study area. (C) Adult specimen of *L*. *arambarensis* from the study area.

*Liolaemus arambarensis* ([47]; Fig 1) is a diurnal, sand-burying lizard from southern Brazil, being one of the two endemic reptiles within the State of Rio Grande do Sul. It occurs in few subtropical restingas (i.e. dry, sandy, sunny areas partially covered by patches of grasses and shrubby plants) at the western margin of the Patos Lagoon, which is the largest barrier-lagoon in South America, located on the Coastal Plain of Rio Grande do Sul (Fig 1). The species is associated with Poaceae grasses, which are mainly used for thermoregulation, predator avoidance and foraging. It is an ambush predator with an omnivorous diet (namely vegetation and arthropods), and presents an oviparous reproductive mode with a well-defined reproductive season between September and March (austral spring and summer). The International Union for Conservation of Nature (IUCN; [48]) lists *L*. *arambarensis* as Endangered. The major factors threatening its persistence are habitat loss and fragmentation, magnified by the lack of new suitable areas to disperse. Only five populations of *L*. *arambarensis* are known to exist, and no individuals have been observed in the last 12 years at the location of the southernmost population [49]. Evidences suggest that populations are genetically isolated due to the lack of continuous sand connections between localities [50].

Field work was conducted in a restinga habitat within the Reserva Particular do Patrimônio Natural Estadual (RPPN) Barba Negra; Celulose Riograndense, municipality of Barra do Ribeiro, Rio Grande do Sul, Brazil (30°24’43”S, 51°13’03”W; 12 m a.s.l.; datum WGS84, Fig 1). It is a private conservation unit with limited human activities and which comprises a spatial niche of sandy areas partially covered by herbaceous and shrubby vegetation, which is adapted to well-drained soils, heat, slight wind and high substrate temperatures resulting from intense solar irradiation [51]. Climate is subtropical humid (type Cfa from Köppen’s classification; mean annual rainfall: 1100–1300 mm; mean annual temperature: 16–18 °C; [52]) with four austral, well-defined seasons. For our study, we selected an area of approximately 1.6 ha with no apparent anthropogenic impact, a relatively high population density [49] and the main habitat features used by the species (vegetated areas, bare sandy areas, windy areas and windless areas). The Celulose Riograndense granted access to the study site.

### Field sampling

We conducted active searches [53] on a monthly basis from January 2013 to December 2016, looking for individuals on two sunny days between 07:00h and 18:00h (BRT). Accordingly, surveying occurred throughout the whole activity period of the species [47]). Each survey was performed along the same spatial transect (approximate length of 500 m), at consistent walking speeds with an equal number of field assistants, in order to maintain standard levels of sampling effort. Active individuals (i.e. moving and not static; see [54]) were found basking or dislocating on the sand surface, within isolated vegetation patches or buried under the sand. After detection, they were captured by hand. Immediately after capture, we recorded the following information: i) *T*_b_, individual’s cloacal temperature; ii) *T*_a_, temperature 5cm above ground level; iii) *T*_s_, temperature at the sand surface and; iv) exact time of detection. Measurements were made using a quick-reading cloacal thermometer Minipa MT-455^®^ (precision 0.1 °C), avoiding direct exposure of the instrument to sunlight. All individuals were marked by toe clipping before release, thus avoiding measurement of the same specimen twice in the same sampling day. Despite certain concerns about this practise, toe clipping is generally accepted for small-sized lizards because of its effectiveness, especially in long-term monitoring, low cost and reduced levels of imposed stress in comparison to other marking techniques, such as pit-tagging [55–57]. Individuals recaptured in different months and years were included in the study since we considered they provided independent information. All research protocols were approved by the Ethics Committee in the Use of Animals from the Universidade Federal do Rio Grande do Sul (CEUA/UFRGS, protocol 22984), and the Chico Mendes Institute for Biodiversity Conservation through the Permit and Information System on Biodiversity (ICMBio/SISBIO, reference 12613–1).

### Data analysis

Records of *T*_b_, *T*_a_ and *T*_s_ (N = 1229 each) were classified according to season and year. Activity observations were defined as the number of active lizards captured for each sampling event (N = 42), and were classified according to the season and year.

To assess *hypothesis 1*, we tested for seasonal and annual differences of activity, *T*_b_, *T*_a_ and *T*_s_, using parametric tests based on our large sample sizes and visual inspections on data; [58]), and we verified the influence of *T*_a_, *T*_s_, season and year on activity and *T*_b_, fitting Generalized Linear Models with normal errors (GLMs; [58]). The thermal environments (i.e. *T*_a_ and *T*_s_) were highly correlated (Pearson’s correlation, r = 0.781, p < 0.001) but were still included in the study and analysed separately, as each may have contributed relevant information for the purpose of the study. Accordingly, we fitted two GLMs–Poisson regression (one including *T*_a_ and the other including *T*_s_) using activity as the response variable, and two GLMs–Gaussian regression with quadratic adjustment (one including *T*_a_ and the other including *T*_s_) using *T*_b_ as the response variable. In every case, we started from models containing all explanatory variables and the interactions between the temporal predictors. Then, we tested for non-significant interactions in order to obtain the most parsimonious models explaining activity and *T*_b_ variations. The Akaike Information Criterion (AIC; [59]) was used to compare the final models fitted for each response variable, in order to determine which thermal environment was more important in explaining the variations. For each model, we evaluated data dispersion and the distribution of residual values.

To assess the relative influence of each thermal environment on *T*_b_ variations, we calculated the difference between *T*_b_ and *T*_a_ (Δ*T*_a_ = *T*_b_−*T*_a_) and between *T*_b_ and *T*_s_ (Δ*T*_*s*_ = *T*_b_−*T*_s_), following the same methodology implemented in other studies of thermal biology conducted on Brazilian reptiles (e.g. [60–62]). Higher absolute values of Δ*T* indicate a higher level of active thermoregulation, using the thermal environment. Although this is not one of the most established indexes of thermoregulation, it allows comparing the relative importance of each thermal environment on *T*_b_ balance across different time scales and, thereby, it contributes to address our study purposes (*hypothesis 2*, *4*).

To test *hypotheses 3* and *4*, relatively mild and harsh seasons were defined according to *T*_a_, using as upper/lower cutoffs the sum/difference between the overall seasonal means and half of the seasonal standard deviations. Annual means of summers/springs and autumns/winters exceeding, respectively, the upper and lower thresholds were considered harsh seasons; conversely, annual means of summers/springs and autumns/winters exceeding, respectively the lower and upper thresholds were considered mild seasons. We tested for significant differences between the *T*_a_ of mild/harsh seasons, using parametric tests. To aide interpretations of the species thermal biology in extreme seasons, we used historical records of seasonal temperatures in the region. For this aim, monthly mean values of maximum, average and minimum temperatures since 1962 (first available records) were recovered from the closest meteorological station (~35 km) to the study site (Porto Alegre–RS; OMM: 83967; 30°05’S, 51°16’W; 47 m a.s.l.; http://www.inmet.gov.br/portal/index.php?r=bdmep/bdmep).

## Results

**Fig 2:**
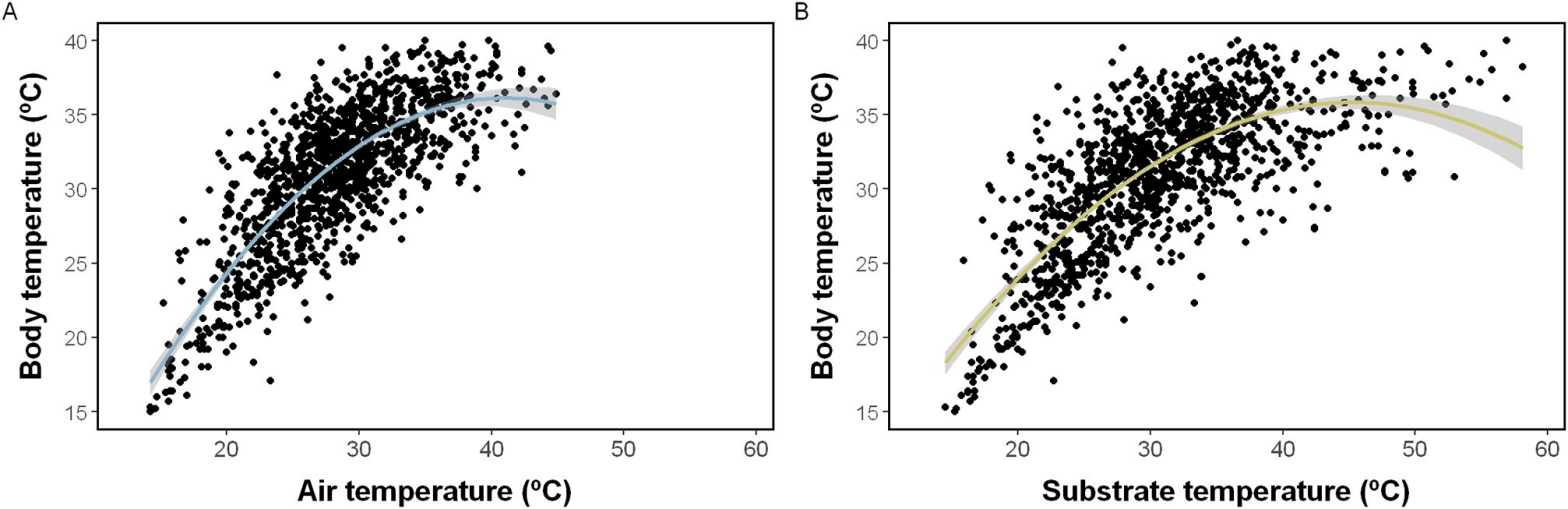
Relationship of *T*_b_ and environmental temperatures. Quadratic relationship (GLM) of body temperature with (A) air temperature (blue) and (B) substrate temperature (yellow), considering each capture of *Liolaemus arambarensis* (N = 1229). The grey strip refers to the confidence interval (95%).

Field surveys totalled 462 hours of sampling effort, wherein 1324 active individuals (69 individuals recaptured two times, seven recaptured three times), were captured and included in the study. Lizards were found active throughout the whole sampling period, at any hour of the day and any moment of the annual cycle. The *T*_b_ exhibited by *L*. *arambarensis* was 31.03 ± 4.90 °C (mean ± standard deviation), ranging within 14.8–43.1 °C, while *T*_a_ and *T*_s_ were respectively 28.29 ± 5.55 °C (12.6–44.9 °C) and 30.76 ± 7.21 °C (14.3–58.1 °C). The thermal environments were positively related to *T*_b_ (Fig 2), and contributed to changes in activity (Table 1) and *T*_b_ (Table 2). Out of these, *T*_a_ was more important in explaining *T*_b_ variations (ΔAIC = 101.7; Table 2), and *T*_s_ in relation to activity variations (ΔAIC = 4.91; Table 1). The Δ*T*_a_ (2.74 ± 3.64 °C) was higher than the Δ*T*_s_ (0.27 ± 5.19 °C; ANOVA: F_1,1227_ = 471.34, p < 0.001).

**Table 1:** Effect of temporal variability and thermal environments on lizards’ activity. GLMs of the effect of seasons and years, and of (A) air temperature or (B) substrate temperature, on the activity of *Liolaemus arambarensis*, considering N = 42 sampling events. Significant results are given in bold.

A	Coefficient	SE	z	p	B	Coefficient	SE	Z	p
*Intercept*	3.44	0.10	33.94	**<2e**^–**16**^	*Intercept*	3.45	0.10	34.08	**<2e**^–**16**^
Air temperature	0.26	0.06	4.06	**4.97e**^–**05**^	Substrate temp.	0.29	0.06	4.62	**3.8e**^–**06**^
Spring	–0.21	0.15	–1.43	0.153	Spring	–0.28	0.15	–1.85	0.064
Summer	0.12	0.15	0.77	0.442	Summer	0.19	0.15	1.24	0.214
Winter	-0.66	0.20	–3.25	**0.001**	Winter	–0.59	0.21	–2.86	**0.004**
2014	0.19	0.14	1.37	0.174	2014	0.14	0.14	0.99	0.321
2015	0.30	0.13	2.26	**0.024**	2015	0.35	0.14	2.57	**0.010**
2016	–0.54	0.19	-2.75	**0.006**	2016	–0.49	0.19	–2.53	**0.012**
Spring:2014	0.10	0.20	0.48	0.632	Spring:2014	0.20	0.20	0.99	0.322
Spring:2015	0.19	0.20	0.97	0.333	Spring:2015	0.17	0.19	0.86	0.392
Spring:2016	0.65	0.25	2.59	**0.010**	Spring:2016	0.56	0.25	2.22	**0.026**
Summer:2014	–0.68	0.23	–2.92	**0.004**	Summer:2014	–0.62	0.23	–2.75	**0.006**
Summer:2015	–0.13	0.23	–0.57	0.569	Summer:2015	–0.30	0.24	–1.24	0.216
Summer:2016	0.30	0.25	1.23	0.219	Summer:2016	0.25	0.25	0.60	0.552
Winter:2014	0.36	0.24	1.49	0.137	Winter:2014	0.23	0.24	0.96	0.336
Winter:2015	0.68	0.25	2.74	**0.006**	Winter:2015	0.54	0.25	2.13	**0.033**
Winter:2016	0.73	0.30	2.40	**0.016**	Winter:2016	0.71	0.30	2.35	**0.019**

Residual deviance: 46.164 (df = 25) | AIC: 297.56 Residual deviance: 41.259 (df = 25) | AIC: 292.65

**Table 2:** Effect of temporal variability and thermal environments on lizards’ *T*_b_. GLMs of the effect of seasons and years, and of (A) air temperature or (B) substrate temperature, on the body temperature of *Liolaemus arambarensis*, considering N = 1228 captures. Significant results are given in bold.

A	Coefficient	SE	t	p	B	Coefficient	SE	T	p
*Intercept*	32.47	0.28	117.28	**<2e**^–**16**^	*Intercept*	32.88	0.29	113.03	**<2e**^**-16**^
Air temperature	3.51	0.10	34.00	**<2e**^–**16**^	Substrate temp.	3.33	0.10	32.00	**<2e**^**-16**^
Air temperature ^2^	–0.67	0.06	–11.23	**<2e**^–**16**^	Substrate temp. ^2^	–0.85	0.06	–15.17	**<2e**^**-16**^
Spring	–0.19	0.40	–0.48	0.632	Spring	0.23	0.41	0.55	0.583
Summer	0.49	0.43	1.15	0.250	Summer	1.15	0.45	2.59	**0.010**
Winter	–0.55	0.54	–1.02	0.306	Winter	–1.00	0.56	–1.78	0.075
2014	–2.41	0.38	–6.28	**4.79e**^–**10**^	2014	–3.27	0.40	–8.21	**5.76e**^–**16**^
2015	–2.56	0.37	–7.02	**3.76e**^–**12**^	2015	–2.78	0.38	–7.34	**3.92e**^–**13**^
2016	–1.82	0.54	–3.36	**8.07e**^–**04**^	2016	–1.55	0.56	–2.79	**0.005**
Spring:2014	2.05	0.56	3.67	**2.50e**^–**04**^	Spring:2014	2.66	0.58	4.59	**5.01e**^–**06**^
Spring:2015	3.41	0.53	6.42	**2.01e**^–**10**^	Spring:2015	2.11	0.55	3.83	**1.35e**^–**04**^
Spring:2016	2.73	0.75	3.66	**2.62e**^–**04**^	Spring:2016	0.86	0.77	1.12	0.265
Summer:2014	1.30	0.62	2.10	**0.036**	Summer:2014	2.59	0.64	4.07	**5.01e**^–**05**^
Summer:2015	0.56	0.63	0.88	0.377	Summer:2015	0.67	0.66	1.02	0.310
Summer:2016	1.74	0.68	2.55	**0.011**	Summer:2016	1.44	0.71	2.02	**0.043**
Winter:2014	1,84	0.66	2.79	**0.005**	Winter:2014	1.19	0.69	1.73	0.083
Winter:2015	2.61	0.67	3.93	**9.12e**^–**05**^	Winter:2015	2.73	0.69	3.94	**8.76e**^–**05**^
Winter:2016	–1.99	0.93	–2.15	**0.032**	Winter:2016	–2.69	0.97	–2.78	**0.005**

Residual deviance: 9012.3 (df = 1210) | AIC: 5970.6 Residual deviance: 9790.5 (df = 1210) | AIC: 6072.3

**Fig 3:**
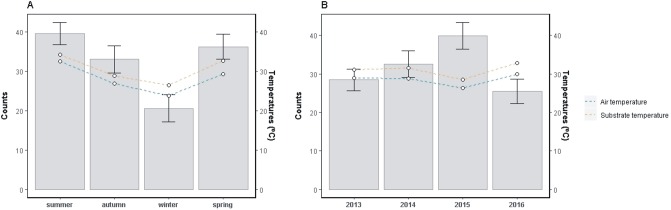
Seasonal and annual variations in activity, in relation to environmental temperatures. Bar charts with error bars show the mean seasonal (A) and annual (B) activity, in relation to mean values of air temperature (blue) and substrate temperature (yellow), considering each capture of *Liolaemus arambarensis* (N = 1324).

**Fig 4:**
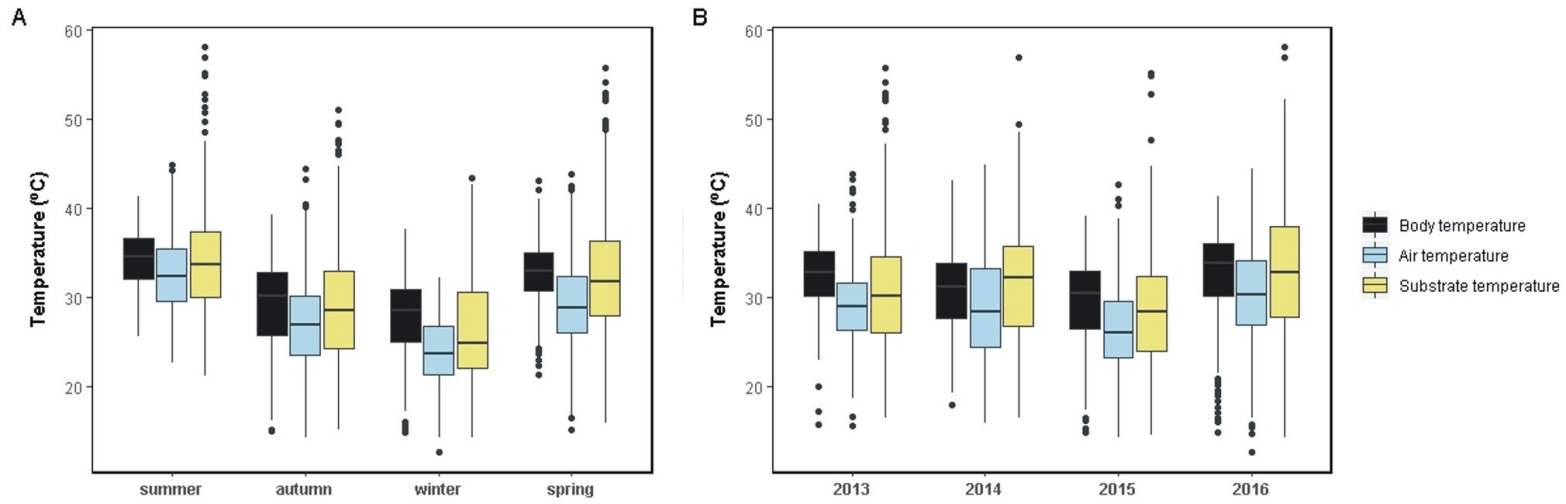
Seasonal and annual variations in *T*_b_ and environmental temperatures. Boxplots show the seasonal (A) and annual (B) variations in body temperature (black), air temperature (blue) and substrate temperature (yellow), considering each capture of *Liolaemus arambarensis* (N = 1229).

**Fig 5:**
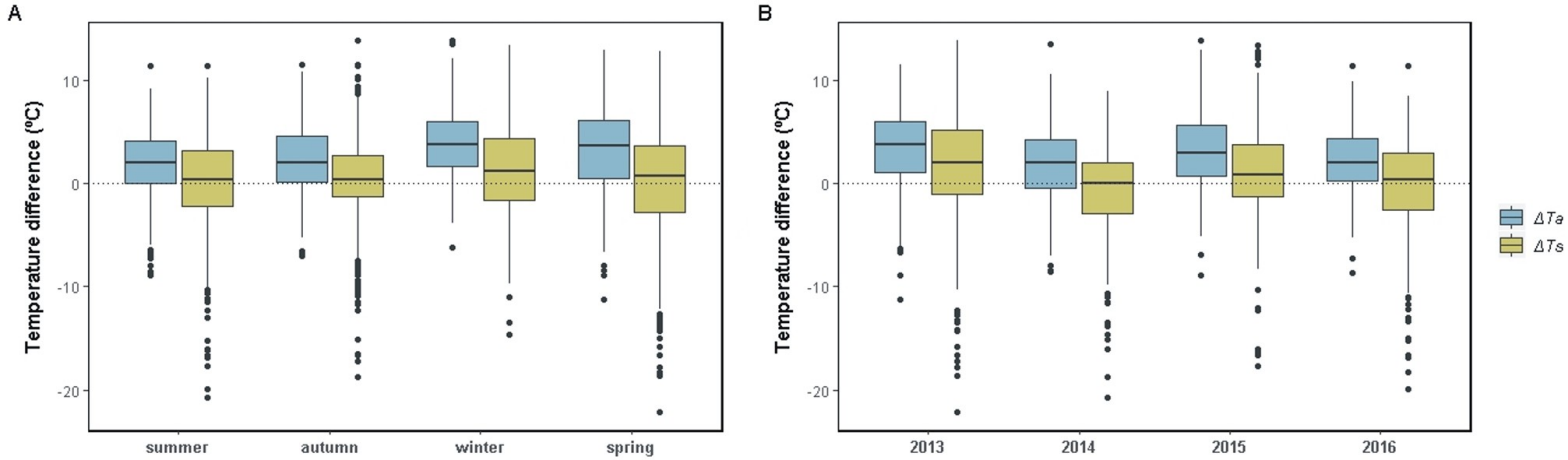
Seasonal and annual variations in active thermoregulation. Boxplots show the differences between body temperature and air temperature (Δ*T*_a_; blue) and between body temperature and substrate temperature (Δ*T*_s_; yellow) for each season (A) and year (B), considering each capture of *Liolaemus arambarensis* (N = 1229).

Lizards’ activity presented peaks during summer and significant drops in winter (Fig 3, S1 Fig, S1 and S2 Tables). Bimodal daily activity patterns with midday shrinks were recorded in spring and summer (S2 Fig). Similarly, *T*_b_ and the thermal environments were warmest during summer, followed by spring, autumn and winter (Fig 4, S1 Fig, S1 and S2 Tables). The Δ*T*_a_ and Δ*T*_s_ were largest in winter and smallest in summer, although differences were strongly significant (p<0.01) only in the former (Fig 5, S1 and S2 Tables). Mean annual values reflected unconnected trends between activity and the thermal environments, as the former was high when the latter were lowest, in 2015 (Fig 3, S1 and S2 Tables). Conversely, inter-annual *T*_b_ variation was consistent with *T*_a_ and *T*_s_, being highest in 2013 and 2016, followed by 2014 and lowest in 2015 (Fig 4, S1 and S2 Tables). The Δ*T*_a_ and Δ*T*_s_ were higher in 2013 and 2015, and lower in 2014 and 2016 (Fig 5, S1 and S2 Tables).

**Fig 6:**
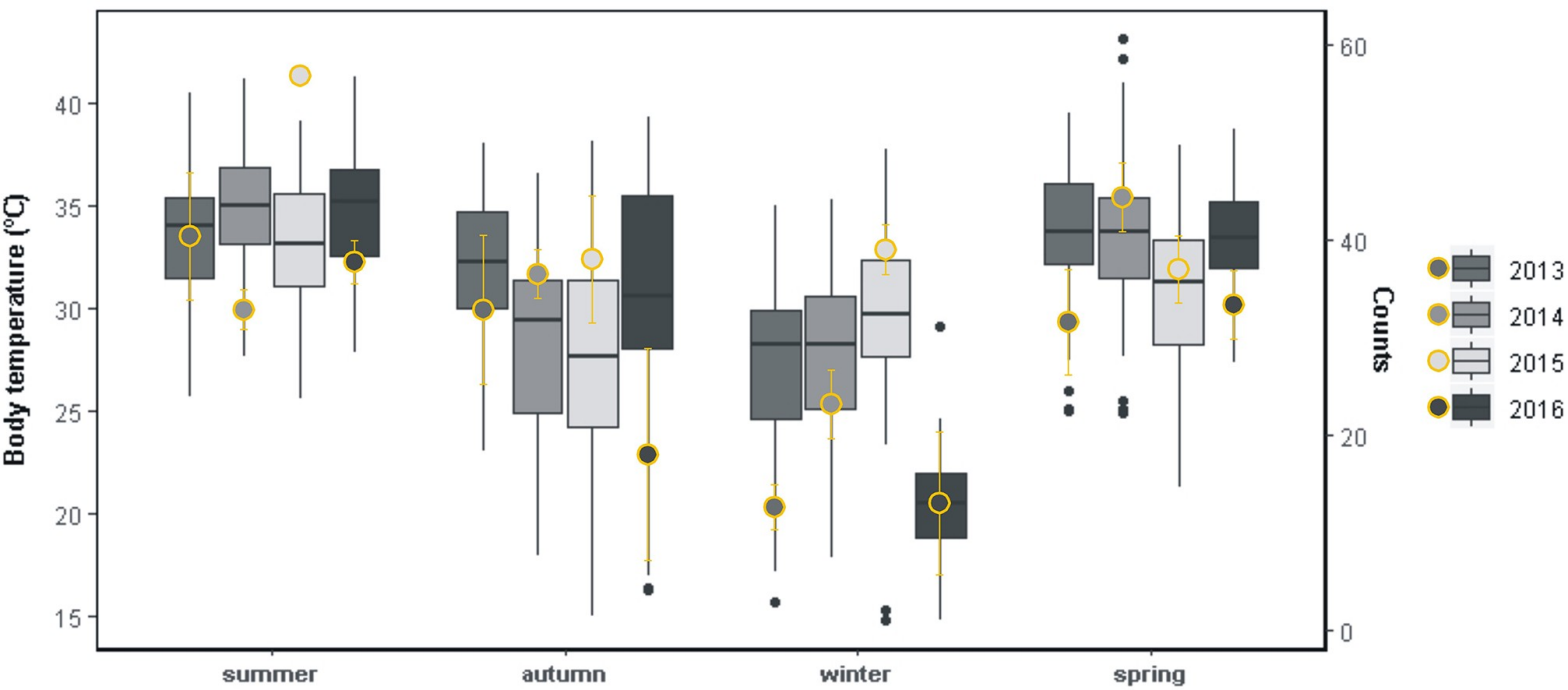
Annual variations in seasonal *T*_b_ and activity. Boxplots show the annual differences in body temperature for each season, considering each capture of *Liolaemus arambarensis* (N = 1229). Yellow points with error bars represent the annual means of seasonal activity, considering each capture of *L*. *arambarensis* (N = 1324). Grey shades represent different years.

Inter-annual shifts in seasonal activity and *T*_b_ (Fig 6, S3 Table) were consistent with the variability of seasonal environmental temperatures (S1 and S3 Figs, S3 and S4 Tables). Mild seasons were summer 2013, spring and winter 2015, and autumn 2016 (S3 and S4 Tables). Significant increases in seasonal activity and *T*_b_ were recorded in the mild winter, with no other deviations revealed but a *T*_b_ decrease in the mild spring (Fig 6, S3 and S4 Tables). Seasonal Δ*T*_a_ and Δ*T*_s_ were greatest, respectively, in the mild summer/spring and in the mild autumn/winter (S4 Fig, S3 Table). Harsh seasons were summer and spring 2014, and winter 2016 (S3 Fig, S3 and S4 Tables). Seasonal activity and Δ*T*_a_ were minimal in the harsh summer while *T*_b_ was conserved (Fig 6, S4 Fig, S3 and S4 Tables), and no changes were observed in the harsh spring (if any, higher seasonal activity). Winter 2016 was extreme (in June: lowest maximum temperature, third lowest average temperature and top-10 lowest minimum temperature ever recorded; S5 Table), matching abrupt decreases in seasonal *T*_b_, activity (Fig 6; S3 Table) and Δ*T*_a_ (S4 Fig, S3 Table). No other extreme seasons were revealed within the study period. The Δ*T*_a_ remained higher than the Δ*T*_s_ in every season within the study period (S4 Fig, S3 Table).

## Discussion

The activity of *L*. *arambarensis* from Barra do Ribeiro extended year-round, although underwent winter limitations. In spite of thermal constraints, sustained winter activity likely responds to various interplaying factors: species distribution transiting between mid and low latitudes, avoiding the cold thresholds which prompt winter dormancy in several temperate lizards [63]; small individual size, resulting in high rates of heat gain/loss and consequently in fast warming-up capacity [64,65]; and habitat features, namely the clear, sandy substrate which acts as a primary heat source in sunny, winter days due to its rapid absorption of solar irradiation, and the dune system that provides windless spots. Winter performance in year-round active species holds a crucial biological role associated to foraging and basking time, which optimises reproductive success through strengthened gonad growth and yield, and embryonic development [31,66,67]. High activity in summer and spring and in midday hours provides evidence for a preference for warm conditions, as expected for an ectotherm, although individuals were often exposed to temperatures of over 40 °C that approach their critical thermal maximum (CT_max_) and jeopardize physiological stability [4,38]. Active thermoregulation, revealed by larger differences between *T*_b_ and the thermal environments at high temperatures, and bimodal activity patterns in summer and spring are common strategies to avoid overheating. Selective microhabitat use, namely midday sheltering in vegetated patches, may also allow evading critical sunlight and sand temperatures while enhancing predator avoidance and foraging [47,68]. The activity patterns of *L*. *arambarensis* are consistent with other lizards dwelling in restingas from southern and southeastern Brazil (e.g. *L*. *occipitalis*, *L*. *lutzae*; [69,70]).

The *T*_b_ of *L*. *arambarensis* showed large amplitude and followed the dynamics of environmental temperatures throughout the annual cycle, as expected in a habitat of marked seasonality. Thermoregulation efficiencies are in accordance with the relationship of *T*_b_ to the environmental temperatures, which are used by the lizards to actively adjust their *T*_b_ [3]. Significant effects of *T*_a_ and *T*_s_ on *T*_b_ were observed, *T*_a_ being better at explaining *T*_b_ variations, and Δ*T*_a_ was higher than Δ*T*_s_ in every season. Our result suggests a heliothermic strategy for *L*. *arambarensis*, which is consistent with the rest of the genus [71]. Results at the seasonal level reveal cold-associated increase in Δ*T*_s_, which points to an expected, greater importance of the sand substrate as a heat source when this is limited; however, correlation of *T*_b_ and *T*_s_ does not necessarily indicate thigmothermy and may just reflect an overall higher need to thermoregulate during cooler periods.

The annual activity maximum matched with the coldest year 2015, although the within-year assessment reveals that the annual lowest temperature was determined by a fresh spring. In addition, summer maximum temperatures stayed below those of 2014 and 2016, and winter was warm. Mild conditions limited lizards’ exposure to their critical thermal limits, likely enhancing activity and facilitating the achievement of optimal temperatures (*T*_o_) with low associated costs [4,39]. The increase in winter activity could have favoured survivorship due to high food intake and reduced thermal constraints, especially in juveniles. The sexual maturity of *L*. *arambarensis* occurs in the breeding season that follows birth, thus survival of young individuals is crucial for reproduction [47]. Although activity may only be weakly connected to demographic trends, the higher levels of 2015 could be translated in population expansion, derived from a strong recruitment of juveniles during an ecologically-favourable breeding period in spring-summer, and high survival rate and strengthened gonad growth and gamete development in winter [31,66,67]. Demographic analyses would enlighten this hypothesis. The other mild seasons within the study period did not differ from the average, although Δ*T*_a_ was high in the fresh summer, as well as in the mild spring, suggesting increased efforts to achieve *T*_o_ during the peak of ecological functions, especially the breeding period.

The thermal physiology of *Liolaemus* lizards is highly labile, where *T*_b_ reflects adaptation to local conditions [43–45]. Accordingly, the *T*_b_ of *L*. *arambarensis* (31.0 °C) differed from other *Liolaemus* species as a function of habitat divergence even for close lineages such as *L*. *salinicola* (36.7 °C), and resembled that of restinga-dwelling species such as *L*. *occipitalis* (30.9 °C) and *L*. *lutzae* (31.7 °C; [44,70,72]). [73] and [70] revealed differences in *Liolaemus T*_b_ also at the intra-specific level. In turn, *T*_pref_ within the genus seem historically conserved within a range of 34–37 °C, and systematically exceed species *T*_b_ [44], especially in subtropical and temperate species; in contrast, [43] pointed to a low phylogenetic conservatism of *T*_pref_. By inference, the operative temperatures (*T*_e_) experienced during activity are ostensibly lower than *T*_pref_. The evolution of lower *T*_b_ likely allowed wider daily and seasonal activity periods in cool environments, increasing foraging time. This may provide *Liolaemus* lizards with a safe physiological margin to buffer global warming [41,44], but subtropical and temperate species may be vulnerable to intense cold spells.

The frigid temperatures of winter 2016, when minimal activity, *T*_b_ and Δ*T* were observed, presumably inhibited activity of *L*. *arambarensis* and hampered thermoregulation to approach *T*_o_. The lowest *T*_b_ records were around 15 °C, staying above the critical thermal minimum (CT_min_) of *Liolaemus* lizards [43]. Yet, active individuals were seldom captured with environmental temperatures below 16–17 °C, supporting a thermal cutoff for activity onset slightly below 20 °C, similar to that proposed by [74] for the closely-related *L*. *wiegmannii* [72]. Winter activity restrictions may imperil population persistence because foraging and basking time is reduced, leading to failed energy intakes that underpin reproductive physiology [31,66,67]. Species range can be also reduced due to activity restrictions, with a drastic effect on microendemisms [15,29,30]. The lability of *Liolaemus* thermal physiology suggests that *L*. *arambarensis* evolved strong specificity to the features of the dune system wherein it is restricted, becoming highly specialized to the local thermal niches. The impact of extreme winter temperatures on activity and *T*_b_ reflects a negative response against adverse conditions, which could entail a major threat due to the restricted distribution of the species [29,30,41]. Although warming is the main climate change-induced effect that impacts the seasonal scale [23,25], recent evidence has also pointed at frigid winters as potentially recurrent events at mid northern latitudes [22,24,75]. The way that the frequency and magnitude of extreme winter spells will change in the study area is uncertain, yet our results suggest that they can induce drastic activity restrictions in *L*. *arambarensis*. In contrast to extreme cold, the conservation of seasonal *T*_b_ in the harsh summer synchronized with relative decreases in activity and Δ*T*_a_ could reflect a cooling response against overheating. However, this pattern was not evident when compared to winter shifts.

Overall, the activity and *T*_b_ of *L*. *arambarensis* aligned with seasonal fluctuations, yet the inter-annual variability of seasonal environmental temperatures shaped their long-term patterns. Fluctuations seem to be especially relevant in winter, due to their apparent high impact favouring/limiting lizards’ performance. Lizards maintain seasonal *T*_b_ in harsh seasons through increasing thermoregulation efforts and even adjusting activity, but extreme cold spells seem to be potentially constraining. Global change impacts local climates in multiple dimensions, including seasonality, which is an underpinning and complex factor that determines the thermal biology of reptiles. Studies of reptiles’ thermal biology generally look at a single year, which potentially overlooks key biological responses to climate variability that can only be unveiled by long-term analytical timeframes.

## Supporting information

S1 Fig Long-term trends in activity, *T*_b_ and environmental temperatures. Evolution of the deviations from the mean values for activity (grey, solid line), body temperature (dark, solid line), air temperature (blue, dashed line), and substrate temperature (yellow, dashed line), throughout the sampling events (N = 42) of *Liolaemus arambarensis*, between January 2013 and December 2016.
(TIFF)

S2 Fig Seasonal daily trends in activity, *T*_b_ and environmental temperatures. Variations in mean activity (grey bars), body temperature (dark, solid line), air temperature (blue, dashed line) and substrate temperature (yellow, dashed line) throughout the day (between 7:00h and 18:00h BTR) for each season, considering each capture of *Liolaemus arambarensis* (N = 1229).
(TIFF)

S3 Fig Annual variations in seasonal environmental temperatures. Boxplots show the annual variations in (A) air temperature and (B) substrate temperature for each season, considering each capture of *Liolaemus arambarensis* (N = 1229).
(TIFF)

S4 Fig Annual variations in seasonal active thermoregulation. Boxplots show the annual differences between body temperature and air temperature (Δ*T*_a_) and between body temperature and substrate temperature (Δ*T*_s_) for each season, considering each capture of *Liolaemus arambarensis* (N = 1229).
(TIFF)

S1 TableMean values in activity, *T*_b_, environmental temperatures and active thermoregulation.Annual and seasonal means and standard deviations ($\bar{x}+\text{sd}$) of activity, body temperature, air temperature, substrate temperature, and differences between body temperature and air temperature (Δ*T*_a_) or substrate (Δ*T*_s_) temperatures, considering each capture of *Liolaemus arambarensis*. Sample sizes are shown in brackets, first for activity and then for temperature data.(DOCX)

S2 Table Seasonal and annual differences in activity, *T*_b_, environmental temperatures and active thermoregulation. P-values of the Tukey HSD post-hoc paired comparisons for activity, body temperature, air temperature, substrate temperature and differences between body temperature and air temperature (Δ*T*_a_) or substrate (Δ*T*_s_) temperatures, between seasons and years. Significant values are given in bold.
(DOCX)

S3 Table Annual mean values in seasonal activity, *T*_b_, environmental temperatures and active thermoregulation. Annual and seasonal means of activity, body temperature, air temperature, substrate temperature and differences between body temperature and air temperature (Δ*T*_a_) or substrate (Δ*T*_s_) temperatures, considering each capture of *Liolaemus arambarensis*. Sample sizes are shown in brackets, first for activity and then for temperature data. Mild/harsh/extreme seasons are depicted, respectively, with green/light-violet/dark-violet.
(DOCX)

S4 Table Differences between normal/mild/harsh seasons. P-values of the Tukey HSD post-hoc paired comparisons for seasonal activity, body temperature and air temperature between years. Significant values are given in bold. Mild/harsh/extreme seasons are depicted, respectively, with green/light-violet/dark-violet.
(DOCX)

S5 Table Historical records of regional temperatures. Monthly mean values of maximum, average and minimum temperatures in the closest meteorological station to the study site (Porto Alegre–RS; OMM: 83967; 30°05’S, 51°16’W).
(DOCX)
