# Supplementary figures and images for: Are lizards sensitive to anomalous seasonal temperatures? Long-term thermobiological variability in a subtropical species
Source: PLoS One. 2019 Dec 19;14(12):e0226399. doi: 10.1371/journal.pone.0226399 (PMC6922334; doi:10.1371/journal.pone.0226399)

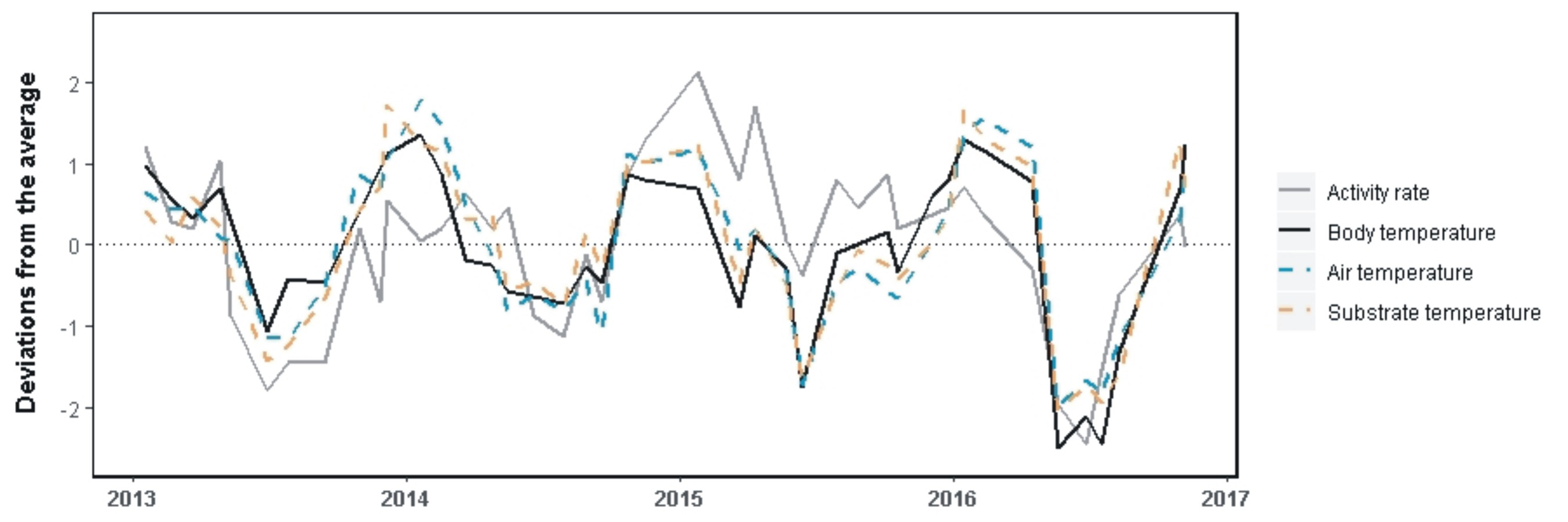

Supplement: S1 Fig — Evolution of the deviations from the mean values for activity (grey, solid line), body temperature (dark, solid line), air temperature (blue, dashed line), and substrate temperature (yellow, dashed line), throughout the sampling events (N = 42) of Liolaemus arambarensis, between January 2013 and December 2016. (TIFF) [file pone.0226399.s001.tiff]

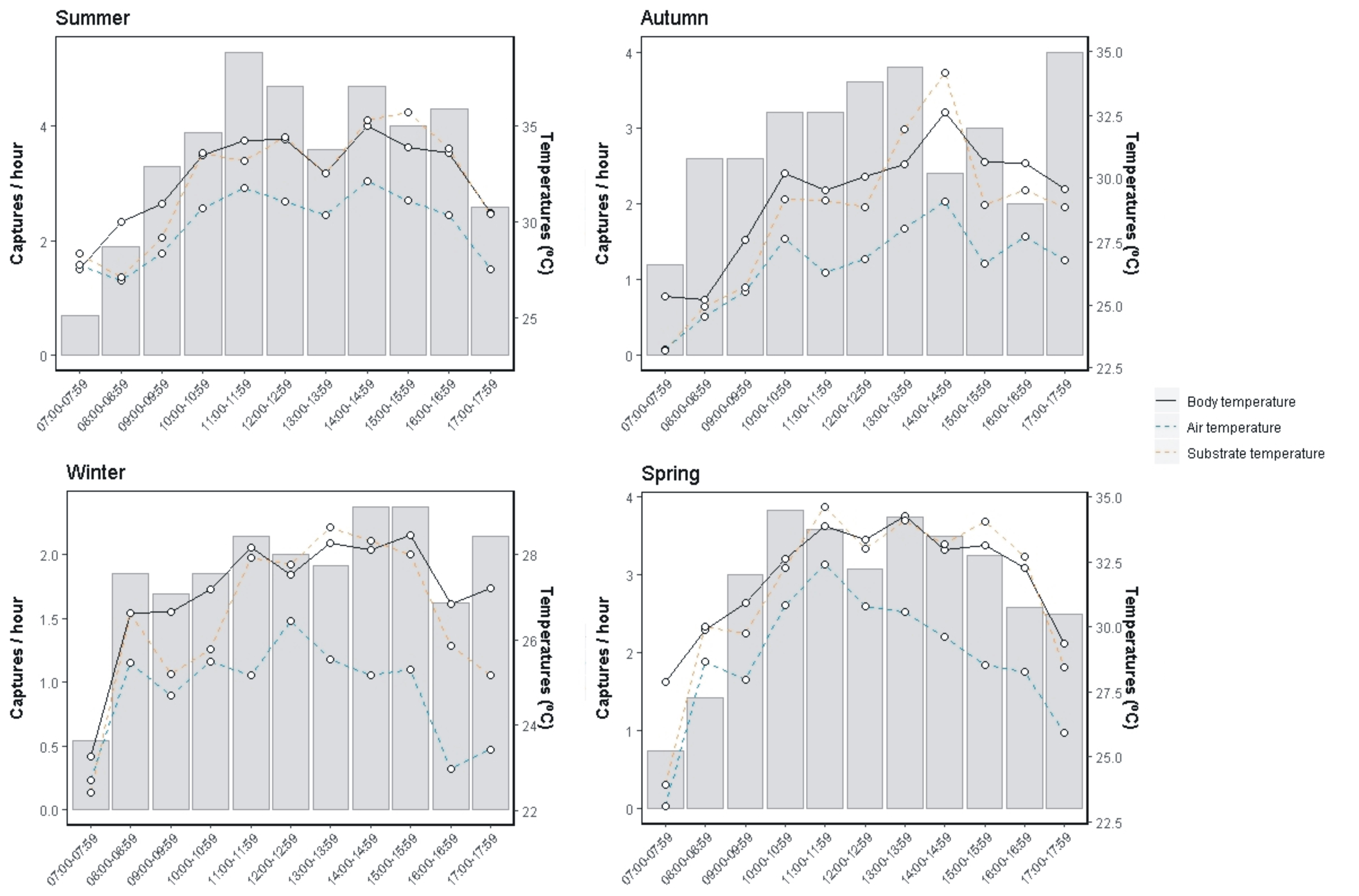

Supplement: S2 Fig — Variations in mean activity (grey bars), body temperature (dark, solid line), air temperature (blue, dashed line) and substrate temperature (yellow, dashed line) throughout the day (between 7:00h and 18:00h BTR) for each season, considering each capture of Liolaemus arambarensis (N = 1229). (TIFF) [file pone.0226399.s002.tiff]

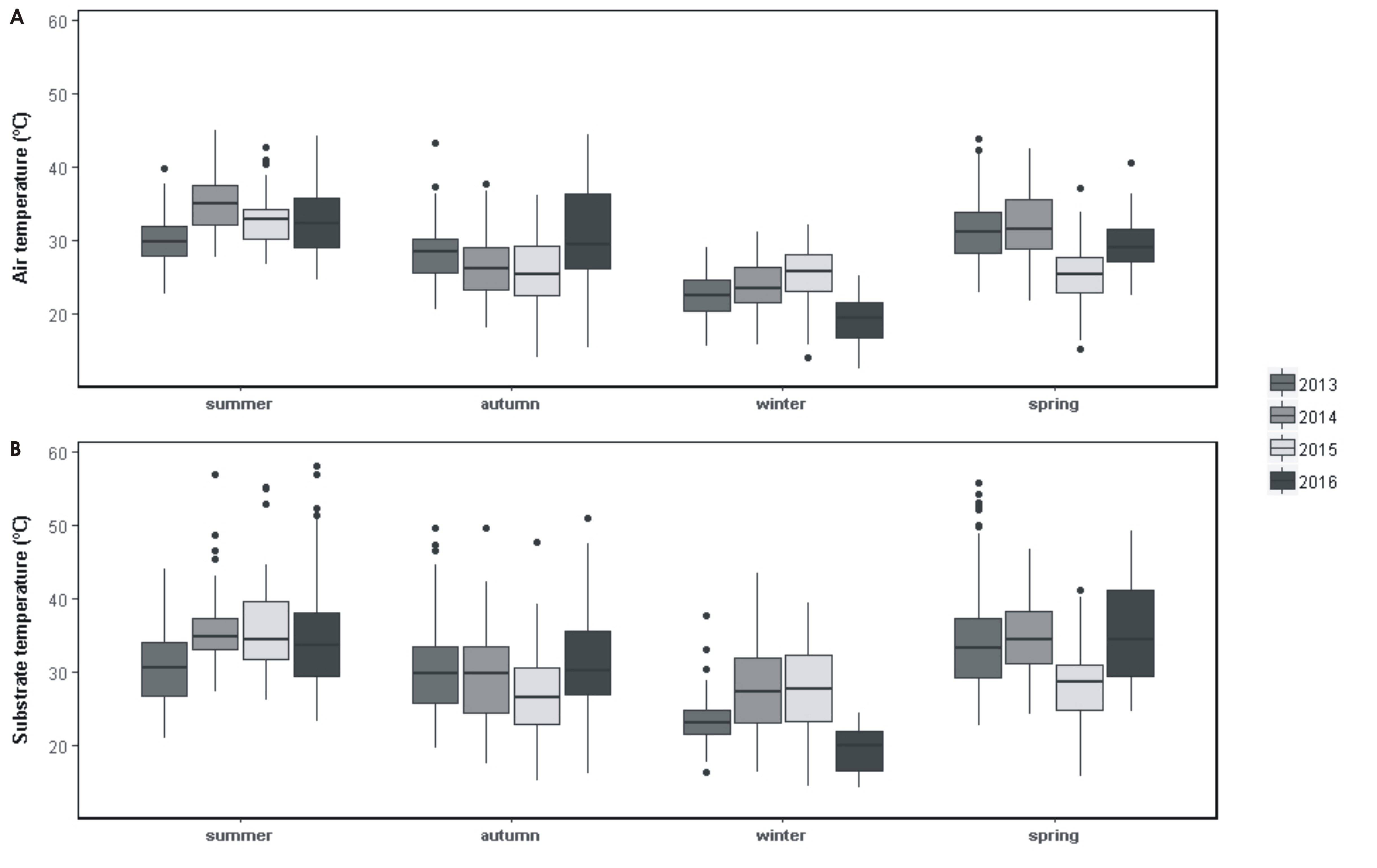

Supplement: S3 Fig — Boxplots show the annual variations in (A) air temperature and (B) substrate temperature for each season, considering each capture of Liolaemus arambarensis (N = 1229). (TIFF) [file pone.0226399.s003.tiff]

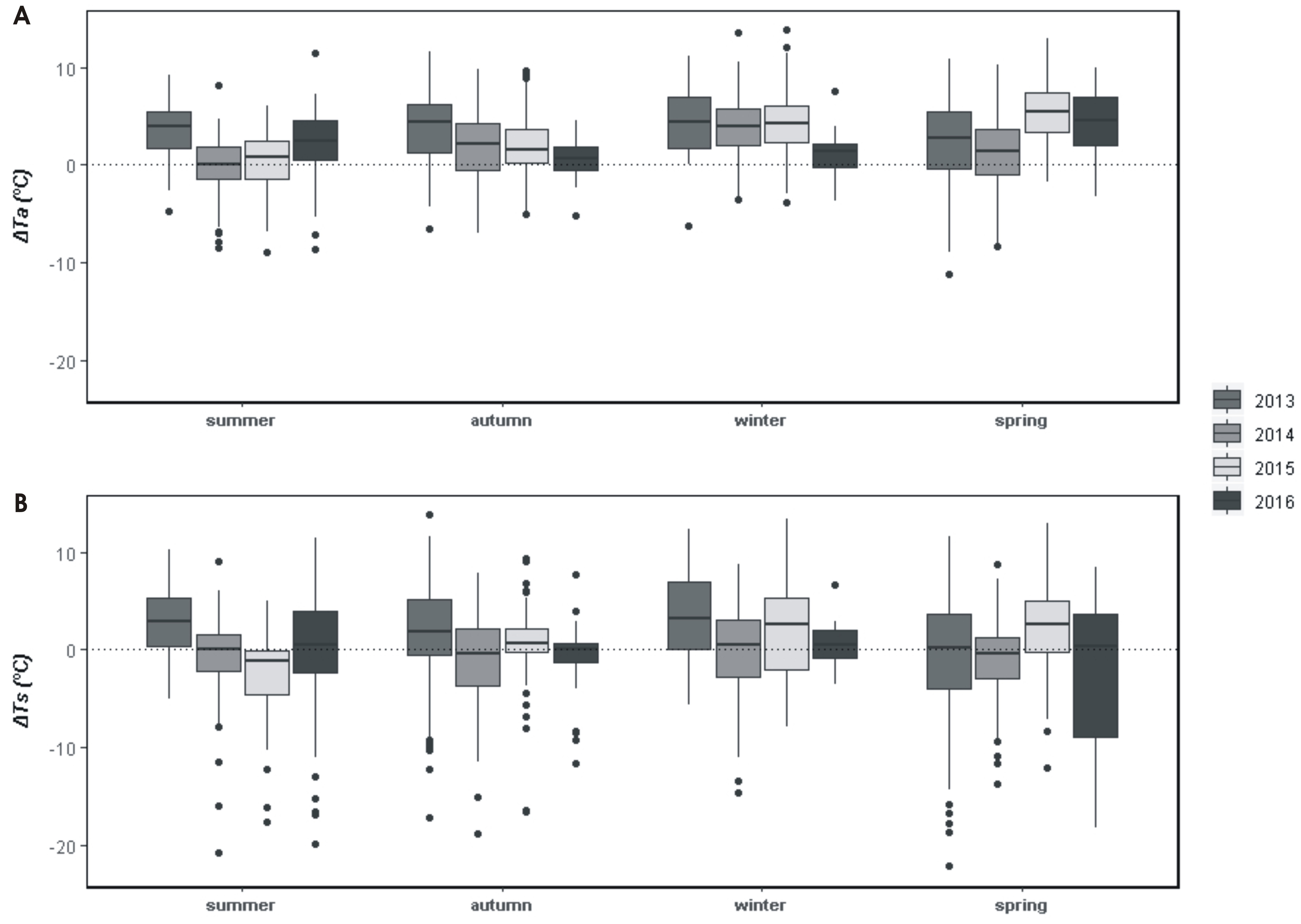

Supplement: S4 Fig — Boxplots show the annual differences between body temperature and air temperature (ΔTa) and between body temperature and substrate temperature (ΔTs) for each season, considering each capture of Liolaemus arambarensis (N = 1229). (TIFF) [file pone.0226399.s004.tiff]
