# Supplementary material for: Are lizards sensitive to anomalous seasonal temperatures? Long-term thermobiological variability in a subtropical species
Source: PLoS One. 2019 Dec 19;14(12):e0226399. doi: 10.1371/journal.pone.0226399 (PMC6922334; doi:10.1371/journal.pone.0226399)
Supplement: S1 Table — Annual and seasonal means and standard deviations (x¯+sd) of activity, body temperature, air temperature, substrate temperature, and differences between body temperature and air temperature (ΔTa) or substrate (ΔTs) temperatures, considering each capture of Liolaemus arambarensis. Sample sizes are shown in brackets, first for activity and then for temperature data. (DOCX) [file pone.0226399.s005.docx]

**SUPPORTING INFORMATION**

**Are lizards sensitive to anomalous seasonal temperatures? Long-term thermobiological variability in a subtropical microendemism**

André Vicente Liz, Vinicius Santos, Talita Ribeiro, Murilo Guimarães, Laura Verrastro

**S1 Table. Mean values in activity, *T*_b_, thermal environment temperatures and active thermoregulation.** Annual and seasonal means and standard deviations (x̄ + sd) of activity, body temperature, air temperature, substrate temperature, and differences between body temperature and air temperature (Δ*T*_a_) or substrate (Δ*T*_s_) temperatures, considering each capture of *Liolaemus arambarensis*. Sample sizes are shown in brackets, first for activity and then for temperature data.

|  | **Activity** (counts) | **Body temp.** (°C) | **Air temp.**  (°C) | **Substrate temp.** (°C) | **Δ*T*_a_**  (°C) | **Δ*T*_s_**  (°C) |
| --- | --- | --- | --- | --- | --- | --- |
| Summer (317; 285) | 39.6 ± 08.2 | 34.24 ± 3.14 | 32.52 ± 4.24 | 34.32 ± 6.41 | 1.72 ± 3.44 | –0.08 ± 5.29 |
| Autumn (397; 381) | 33.1 ± 11.4 | 29.17 ± 5.03 | 26.87 ± 5.09 | 28.92 ± 6.61 | 2.30 ± 3.27 | 0.25 ± 4.54 |
| Winter (247; 228) | 20.6 ± 11.3 | 27.71 ± 4.60 | 23.78 ± 3.80 | 26.45 ± 6.07 | 3.93 ± 3.36 | 1.26 ± 4.79 |
| Spring (363; 335) | 36.3 ± 06.7 | 32.67 ± 3.68 | 29.38 ± 5.10 | 32.75 ± 7.07 | 3.29 ± 4.08 | –0.08 ± 5.94 |
| 2013 (313; 300) | 28.5 ± 12.6 | 32.38 ± 3.93 | 28.96 ± 4.58 | 31.12 ± 7.19 | 3.41 ± 3.67 | 1.26 ± 5.84 |
| 2014 (357; 352) | 32.5 ± 09.0 | 30.76 ± 4.78 | 28.84 ± 5.96 | 31.59 ± 6.48 | 1.92 ± 3.72 | –0.83 ± 4.59 |
| 2015 (399; 377) | 39.9 ± 09.0 | 29.58 ± 4.71 | 26.37 ± 4.91 | 28.56 ± 6.50 | 3.22 ± 3.56 | 1.02 ± 4.59 |
| 2016 (255; 200) | 25.5 ± 13.4 | 32.20 ± 5.86 | 29.93 ± 6.28 | 32.88 ± 8.61 | 2.26 ± 3.29 | –0.69 ± 5.69 |
