# Supplementary material for: Are lizards sensitive to anomalous seasonal temperatures? Long-term thermobiological variability in a subtropical species
Source: PLoS One. 2019 Dec 19;14(12):e0226399. doi: 10.1371/journal.pone.0226399 (PMC6922334; doi:10.1371/journal.pone.0226399)
Supplement: S2 Table — P-values of the Tukey HSD post-hoc paired comparisons for activity, body temperature, air temperature, substrate temperature and differences between body temperature and air temperature (ΔTa) or substrate (ΔTs) temperatures, between seasons and years. Significant values are given in bold. (DOCX) [file pone.0226399.s006.docx]

**SUPPORTING INFORMATION**

**Are lizards sensitive to anomalous seasonal temperatures? Long-term thermobiological variability in a subtropical microendemism**

André Vicente Liz, Vinicius Santos, Talita Ribeiro, Murilo Guimarães, Laura Verrastro

**S2 Table. Seasonal and annual differences in activity, *T*_b_, thermal environment temperatures and active thermoregulation.** P-values of the Tukey HSD post-hoc paired comparisons for activity, body temperature, air temperature, substrate temperature and differences between body temperature and air temperature (Δ*T*_a_) or substrate (Δ*T*_s_) temperatures, between seasons and years. Significant values are given in bold.

| **Activity** | Season | Summer | Autumn | Winter | Spring | Year | 2013 | 2014 | 2015 | 2016 |
| --- | --- | --- | --- | --- | --- | --- | --- | --- | --- | --- |
|  | Summer |  |  |  |  | 2013 |  |  |  |  |
|  | Autumn | 0.476 |  |  |  | 2014 | 0.835 |  |  |  |
|  | Winter | **<0.001** | **0.018** |  |  | 2015 | 0.106 | 0.433 |  |  |
|  | Spring | 0.893 | 0.871 | **0.003** |  | 2016 | 0.930 | 0.492 | **0.031** |  |
| **Body temp.** | Season | Summer | Autumn | Winter | Spring | Year | 2013 | 2014 | 2015 | 2016 |
|  | Summer |  |  |  |  | 2013 |  |  |  |  |
|  | Autumn | **<0.001** |  |  |  | 2014 | **<0.001** |  |  |  |
|  | Winter | **<0.001** | **<0.001** |  |  | 2015 | **<0.001** | **0.005** |  |  |
|  | Spring | **<0.001** | **<0.001** | **<0.001** |  | 2016 | 0.978 | **0.004** | **<0.001** |  |
| **Air temp.** | Season | Summer | Autumn | Winter | Spring | Year | 2013 | 2014 | 2015 | 2016 |
|  | Summer |  |  |  |  | 2013 |  |  |  |  |
|  | Autumn | **<0.001** |  |  |  | 2014 | 0.992 |  |  |  |
|  | Winter | **<0.001** | **<0.001** |  |  | 2015 | **<0.001** | **<0.001** |  |  |
|  | Spring | **<0.001** | **<0.001** | **<0.001** |  | 2016 | 0.200 | 0.102 | **<0.001** |  |
| **Substr.ate temp.** | Season | Summer | Autumn | Winter | Spring | Year | 2013 | 2014 | 2015 | 2016 |
|  | Summer |  |  |  |  | 2013 |  |  |  |  |
|  | Autumn | **<0.001** |  |  |  | 2014 | 0.829 |  |  |  |
|  | Winter | **<0.001** | **<0.001** |  |  | 2015 | **<0.001** | **<0.001** |  |  |
|  | Spring | 0.**017** | **<0.001** | **<0.001** |  | 2016 | 0.317 | 0.164 | **<0.001** |  |
| **Δ*T*_a_** | Season | Summer | Autumn | Winter | Spring | Year | 2013 | 2014 | 2015 | 2016 |
|  | Summer |  |  |  |  | 2013 |  |  |  |  |
|  | Autumn | 0.160 |  |  |  | 2014 | **<0.001** |  |  |  |
|  | Winter | **<0.001** | **<0.001** |  |  | 2015 | 0.892 | **<0.001** |  |  |
|  | Spring | **<0.001** | **0.001** | 0.155 |  | 2016 | **0.002** | 0.702 | **0.013** |  |
| **Δ*T*_s_** | Season | Summer | Autumn | Winter | Spring | Year | 2013 | 2014 | 2015 | 2016 |
|  | Summer |  |  |  |  | 2013 |  |  |  |  |
|  | Autumn | 0.843 |  |  |  | 2014 | **<0.001** |  |  |  |
|  | Winter | **0.019** | 0.092 |  |  | 2015 | 0.933 | **<0.001** |  |  |
|  | Spring | 1.000 | 0.825 | **0.014** |  | 2016 | **<0.001** | 0.989 | **<0.001** |  |
