# Supplementary material for: Are lizards sensitive to anomalous seasonal temperatures? Long-term thermobiological variability in a subtropical species
Source: PLoS One. 2019 Dec 19;14(12):e0226399. doi: 10.1371/journal.pone.0226399 (PMC6922334; doi:10.1371/journal.pone.0226399)
Supplement: S4 Table — P-values of the Tukey HSD post-hoc paired comparisons for seasonal activity, body temperature and air temperature between years. Significant values are given in bold. Mild/harsh/extreme seasons are depicted, respectively, with green/light-violet/dark-violet. (DOCX) [file pone.0226399.s008.docx]

**SUPPORTING INFORMATION**

**Are lizards sensitive to anomalous seasonal temperatures? Long-term thermobiological variability in a subtropical microendemism**

André Vicente Liz, Vinicius Santos, Talita Ribeiro, Murilo Guimarães, Laura Verrastro

**S4 Table. Differences between normal/mild/harsh seasons.** P-values of the Tukey HSD post-hoc paired comparisons for seasonal activity, body temperature and air temperature between years. Significant values are given in bold. Mild/harsh/extreme seasons are depicted, respectively, with green/light-violet/dark-violet.

| **Activity** | | | | | | | | | |
| --- | --- | --- | --- | --- | --- | --- | --- | --- | --- |
| Summer | 2013 | 2014 | 2015 | 2016 | Autumn | 2013 | 2014 | 2015 | 2016 |
| 2013 |  |  |  |  | 2013 |  |  |  |  |
| 2014 | 0.398 |  |  |  | 2014 | 0.970 |  |  |  |
| 2015 | 0.104 | **0.032** |  |  | 2015 | 0.917 | 0.998 |  |  |
| 2016 | 0.878 | 0.652 | 0.054 |  | 2016 | 0.429 | 0.266 | 0.189 |  |
| Winter | 2013 | 2014 | 2015 | 2016 | Spring | 2013 | 2014 | 2015 | 2016 |
| 2013 |  |  |  |  | 2013 |  |  |  |  |
| 2014 | 0.238 |  |  |  | 2014 | 0.145 |  |  |  |
| 2015 | **0.009** | 0.083 |  |  | 2015 | 0.613 | 0.526 |  |  |
| 2016 | 1.000 | 0.260 | **0.010** |  | 2016 | 0.982 | 0.283 | 0.867 |  |
| **Body temperature** | | | | | | | | | |
| Summer | 2013 | 2014 | 2015 | 2016 | Autumn | 2013 | 2014 | 2015 | 2016 |
| 2013 |  |  |  |  | 2013 |  |  |  |  |
| 2014 | 0.170 |  |  |  | 2014 | **<0.001** |  |  |  |
| 2015 | 0.757 | **0.025** |  |  | 2015 | **<0.001** | 0.297 |  |  |
| 2016 | 0.140 | 0.999 | **0.017** |  | 2016 | 0.123 | 0.256 | **0.011** |  |
| Winter | 2013 | 2014 | 2015 | 2016 | Spring | 2013 | 2014 | 2015 | 2016 |
| 2013 |  |  |  |  | 2013 |  |  |  |  |
| 2014 | 0.909 |  |  |  | 2014 | 0.999 |  |  |  |
| 2015 | **0.009** | **0.007** |  |  | 2015 | **<0.001** | **<0.001** |  |  |
| 2016 | **<0.001** | **<0.001** | **<0.001** |  | 2016 | 0.970 | 0.985 | **<0.001** |  |
| **Air temperature** | | | | | | | | | |
| Summer | 2013 | 2014 | 2015 | 2016 | Autumn | 2013 | 2014 | 2015 | 2016 |
| 2013 |  |  |  |  | 2013 |  |  |  |  |
| 2014 | **<0.001** |  |  |  | 2014 | **0.002** |  |  |  |
| 2015 | **<0.001** | **0.003** |  |  | 2015 | **<0.001** | 0.223 |  |  |
| 2016 | **<0.001** | **<0.001** | 0.600 |  | 2016 | 0.119 | **0.007** | **0.001** |  |
| Winter | 2013 | 2014 | 2015 | 2016 | Spring | 2013 | 2014 | 2015 | 2016 |
| 2013 |  |  |  |  | 2013 |  |  |  |  |
| 2014 | 0.171 |  |  |  | 2014 | 0.199 |  |  |  |
| 2015 | **<0.001** | **0.002** |  |  | 2015 | **<0.001** | **<0.001** |  |  |
| 2016 | **<0.001** | **<0.001** | **<0.001** |  | 2016 | **0.006** | **<0.001** | **<0.001** |  |
