# Supplementary material for: Are lizards sensitive to anomalous seasonal temperatures? Long-term thermobiological variability in a subtropical species
Source: PLoS One. 2019 Dec 19;14(12):e0226399. doi: 10.1371/journal.pone.0226399 (PMC6922334; doi:10.1371/journal.pone.0226399)
Supplement: S5 Table — Monthly mean values of maximum, average and minimum temperatures in the closest meteorological station to the study site (Porto Alegre–RS; OMM: 83967; 30°05’S, 51°16’W). (DOCX) [file pone.0226399.s009.docx]

**SUPPORTING INFORMATION**

**Are lizards sensitive to anomalous seasonal temperatures? Long-term thermobiological variability in a subtropical microendemism**

André Vicente Liz, Vinicius Santos, Talita Ribeiro, Murilo Guimarães, Laura Verrastro

**S5 Table. Historical records of regional temperatures.** Monthly mean values of maximum, average and minimum temperatures in the closest meteorological station to the study site (Porto Alegre – RS; OMM: 83967; 30°05’S, 51°16’W).

| **Month** | **Maximum**  **temperature (**°C) | **Average**  **temperature (**°C) | **Minimum**  **temperature (**°C) |
| --- | --- | --- | --- |
| 01/1962 | 29,25 | 24 | 19,75 |
| 02/1962 | 28,41 | 22,96 | 18,76 |
| 03/1962 | 29,94 | 23,74 | 19,67 |
| 04/1962 | 24,75 | 19,32 | 15,3 |
| 05/1962 | 20,7 | 15,07 | 11,08 |
| 06/1962 | 20,58 | 13,86 | 9,4 |
| 07/1962 | 17,05 | 12,79 | 10,02 |
| 08/1962 | 20,12 | 14,25 | 10,06 |
| 09/1962 | 22,58 | 17,05 | 12,91 |
| 10/1962 | 23,7 | 18,01 | 13,59 |
| 11/1962 | 27,92 | 22,03 | 17,49 |
| 12/1962 | 30,63 | 24,2 | 19,43 |
| 01/1963 | 30,1 | 24,95 | 21,37 |
| 02/1963 | 30,43 | 25,01 | 21,25 |
| 03/1963 | 30 | 24,23 | 20,49 |
| 04/1963 | 26,96 | 20,41 | 16,1 |
| 05/1963 | 23,08 | 17,22 | 13,43 |
| 06/1963 | 20,56 | 15,01 | 10,86 |
| 07/1963 | 21,65 | 15,42 | 11,44 |
| 08/1963 | 19,3 | 14,8 | 11,6 |
| 09/1963 | 21,9 | 18,01 | 15,51 |
| 10/1963 | 23,08 | 19,04 | 15,74 |
| 11/1963 | 26,26 | 21,16 | 17,41 |
| 12/1963 | 28,63 | 23,04 | 18,89 |
| 01/1964 | 30,86 | 24,58 | 19,93 |
| 02/1964 | 29,59 | 24,33 | 20,44 |
| 03/1964 | 28,76 | 23,31 | 19,37 |
| 04/1964 | 25,38 | 20,61 | 17,35 |
| 05/1964 | 22,72 | 17,03 | 13,41 |
| 06/1964 | 18,37 | 12,45 | 8,33 |
| 07/1964 | 17,67 | 12,18 | 8,27 |
| 08/1964 | 20,63 | 15,44 | 11,61 |
| 09/1964 | 21,29 | 16,56 | 13,06 |
| 10/1964 | 22,96 | 18 | 14,17 |
| 11/1964 | 26,16 | 20,4 | 15,72 |
| 12/1964 | 28,4 | 22,26 | 17,61 |
| 01/1965 | 30,35 | 24,47 | 19,88 |
| 02/1965 | 31,64 | 25,53 | 21,14 |
| 03/1965 | 26,28 | 21,22 | 17,63 |
| 04/1965 | 24,58 | 19,68 | 16,05 |
| 05/1965 | 20,94 | 15,78 | 12,15 |
| 06/1965 | 21,6 | 16,29 | 12,86 |
| 07/1965 | 19,28 | 13,91 | 10,01 |
| 08/1965 | 20,29 | 15,69 | 12,24 |
| 09/1965 | 21,18 | 16,77 | 13,22 |
| 10/1965 | 25,23 | 19,78 | 15,6 |
| 11/1965 | 26,92 | 21,65 | 17,66 |
| 12/1965 | 28,54 | 23,53 | 19,69 |
| 01/1966 | 29,84 | 24,72 | 20,86 |
| 02/1966 | 28,79 | 23,97 | 20,68 |
| 03/1966 | 28,08 | 22,98 | 19,03 |
| 04/1966 | 25,58 | 20,85 | 17,64 |
| 05/1966 | 23,57 | 17,34 | 13,15 |
| 06/1966 | 21,12 | 15,7 | 12,22 |
| 07/1966 | 20,21 | 14,95 | 11,29 |
| 08/1966 | 18,68 | 13,5 | 9,76 |
| 09/1966 | 20,08 | 15,68 | 12,32 |
| 10/1966 | 23,5 | 18,64 | 14,97 |
| 11/1966 | 27,37 | 21,76 | 17,55 |
| 12/1966 | 28,24 | 23,24 | 19,52 |
| 01/1967 | 29,71 | 24,15 | 19,94 |
| 02/1967 | 28,99 | 23,87 | 20,23 |
| 03/1967 | 27,23 | 21,94 | 18,08 |
| 04/1967 | 25,69 | 19,87 | 15,91 |
| 05/1967 | 25,04 | 19,65 | 16,33 |
| 07/1967 | 20,77 | 15,22 | 11,57 |
| 08/1967 | 22,47 | 16,7 | 12,76 |
| 09/1967 | 22,07 | 17,91 | 14,56 |
| 10/1967 | 26,55 | 21,55 | 17,82 |
| 11/1967 | 26,6 | 21,71 | 17,72 |
| 12/1967 | 31,04 | 23,95 | 18,29 |
| 01/1968 | 30,64 | 24,63 | 20,36 |
| 02/1968 | 31,12 | 24,54 | 19,43 |
| 03/1968 | 28,94 | 23,45 | 19,58 |
| 04/1968 | 22,67 | 16,92 | 12,68 |
| 05/1968 | 20,6 | 14,11 | 9,64 |
| 06/1968 | 19,95 | 14,72 | 11,3 |
| 07/1968 | 20,93 | 15,35 | 11,52 |
| 08/1968 | 21,96 | 16,32 | 12 |
| 09/1968 | 22,47 | 16,75 | 12,13 |
| 10/1968 | 24,85 | 19,57 | 15,4 |
| 11/1968 | 29,16 | 23,52 | 19,15 |
| 12/1968 | 29,75 | 23,97 | 19,36 |
| 01/1969 | 29,37 | 23,79 | 19,7 |
| 02/1969 | 30,08 | 24,71 | 20,85 |
| 03/1969 | 28,41 | 23,01 | 19,4 |
| 04/1969 | 25,95 | 20,63 | 16,79 |
| 05/1969 | 23,6 | 17,65 | 13,64 |
| 06/1969 | 20,22 | 15,25 | 11,53 |
| 07/1969 | 20,82 | 14,58 | 10,16 |
| 08/1969 | 19,86 | 14,16 | 10,05 |
| 09/1969 | 23,14 | 17,21 | 12,87 |
| 10/1969 | 24,03 | 18,09 | 13,65 |
| 11/1969 | 26,77 | 21,45 | 17,47 |
| 12/1969 | 29,63 | 23,2 | 18,32 |
| 01/1970 | 31,18 | 25,1 | 20,83 |
| 02/1970 | 31,64 | 25,71 | 21,45 |
| 03/1970 | 29,37 | 24,13 | 20,35 |
| 04/1970 | 28,04 | 21,57 | 16,77 |
| 05/1970 | 22,93 | 18,01 | 14,72 |
| 06/1970 | 20,35 | 15,37 | 11,83 |
| 07/1970 | 20,34 | 14,84 | 11,07 |
| 08/1970 | 20,95 | 15,29 | 10,93 |
| 09/1970 | 22,97 | 17,45 | 13,18 |
| 10/1970 | 23,08 | 18,58 | 14,94 |
| 11/1970 | 25,26 | 19,55 | 15,16 |
| 12/1970 | 29,9 | 23,76 | 19,06 |
| 01/1971 | 31,83 | 25,51 | 21,29 |
| 02/1971 | 30,6 | 25,19 | 21,31 |
| 03/1971 | 27,66 | 22,79 | 19,37 |
| 04/1971 | 23,88 | 18,49 | 14,54 |
| 05/1971 | 20,55 | 15,01 | 11,37 |
| 06/1971 | 16,96 | 12,54 | 9,1 |
| 07/1971 | 20,02 | 14,74 | 11,26 |
| 08/1971 | 20,7 | 15,43 | 11,55 |
| 09/1971 | 24,83 | 19,61 | 15,92 |
| 10/1971 | 25,55 | 19,61 | 15,17 |
| 11/1971 | 27,78 | 21,71 | 16,97 |
| 12/1971 | 31,46 | 24,56 | 19,21 |
| 01/1972 | 30,28 | 24,53 | 20,2 |
| 02/1972 | 29,12 | 23,83 | 19,56 |
| 03/1972 | 27,56 | 22,87 | 19,52 |
| 04/1972 | 24,46 | 19,21 | 15,32 |
| 05/1972 | 24,2 | 19,17 | 15,51 |
| 06/1972 | 21,33 | 17,85 | 15,37 |
| 07/1972 | 19,98 | 14,72 | 10,99 |
| 08/1972 | 19,65 | 15,22 | 12,12 |
| 09/1972 | 22,8 | 17,51 | 13,35 |
| 10/1972 | 23,33 | 18,42 | 14,81 |
| 11/1972 | 24,95 | 20,47 | 17,04 |
| 12/1972 | 29,65 | 24,09 | 19,77 |
| 01/1973 | 30,5 | 25,27 | 21,44 |
| 02/1973 | 29,81 | 25,14 | 21,83 |
| 03/1973 | 28,27 | 23,22 | 19,38 |
| 04/1973 | 26,52 | 21,87 | 18,55 |
| 05/1973 | 22,03 | 17,77 | 14,66 |
| 06/1973 | 20,48 | 15,98 | 13,01 |
| 07/1973 | 19,5 | 14,95 | 11,99 |
| 08/1973 | 18,21 | 13,5 | 9,96 |
| 09/1973 | 20,76 | 16,17 | 12,51 |
| 10/1973 | 24,49 | 19,42 | 15,55 |
| 11/1973 | 26,24 | 20,48 | 15,52 |
| 12/1973 | 29,12 | 23,77 | 19,87 |
| 01/1974 | 31,35 | 25,67 | 21,1 |
| 02/1974 | 29,46 | 24,73 | 21,42 |
| 03/1974 | 27,78 | 23,31 | 19,82 |
| 04/1974 | 26,07 | 20,55 | 16,47 |
| 05/1974 | 23,35 | 18,23 | 14,57 |
| 06/1974 | 17,72 | 12,92 | 9,94 |
| 07/1974 | 21,24 | 15,74 | 12,51 |
| 08/1974 | 21,01 | 14,8 | 10,3 |
| 09/1974 | 21,49 | 15,99 | 12,25 |
| 10/1974 | 23,33 | 17,65 | 13,12 |
| 11/1974 | 25,79 | 19,98 | 15,47 |
| 12/1974 | 26,47 | 21,33 | 17,45 |
| 01/1975 | 28,71 | 22,96 | 18,24 |
| 02/1975 | 29,03 | 23,76 | 19,65 |
| 03/1975 | 27,63 | 22,74 | 19,19 |
| 04/1975 | 24,73 | 19,63 | 16,08 |
| 05/1975 | 22,15 | 16,89 | 12,49 |
| 06/1975 | 19,54 | 14,69 | 10,99 |
| 07/1975 | 18,63 | 13,48 | 9,7 |
| 08/1975 | 20,2 | 15,81 | 12,52 |
| 09/1975 | 21,53 | 17,04 | 13,84 |
| 10/1975 | 22,71 | 18,05 | 14,39 |
| 11/1975 | 25,9 | 20,47 | 16,15 |
| 12/1975 | 28,78 | 23,05 | 18,27 |
| 01/1976 | 29,65 | 24,42 | 20,61 |
| 02/1976 | 28,88 | 23,55 | 19,41 |
| 03/1976 | 26,63 | 21,36 | 17,13 |
| 04/1976 | 23,61 | 18,4 | 14,8 |
| 05/1976 | 20,69 | 16,46 | 13,55 |
| 06/1976 | 17,9 | 13,13 | 9,56 |
| 07/1976 | 18,62 | 13,89 | 10,36 |
| 08/1976 | 18,99 | 14,15 | 10,39 |
| 09/1976 | 21,34 | 15,87 | 11,91 |
| 10/1976 | 23,55 | 18,16 | 14,09 |
| 11/1976 | 25,44 | 20,15 | 16,03 |
| 12/1976 | 28,87 | 23,17 | 18,45 |
| 01/1977 | 30,08 | 24,87 | 21,2 |
| 02/1977 | 32,08 | 25,84 | 22,08 |
| 03/1977 | 28,19 | 23,32 | 19,72 |
| 04/1977 | 24,06 | 19,34 | 15,54 |
| 05/1977 | 20,57 | 16,14 | 12,81 |
| 06/1977 | 19,69 | 15,12 | 12,06 |
| 07/1977 | 22,46 | 17,03 | 13,11 |
| 08/1977 | 18,91 | 14,71 | 11,8 |
| 09/1977 | 24,63 | 19,03 | 15,15 |
| 10/1977 | 26,76 | 21,17 | 17,09 |
| 11/1977 | 27,08 | 21,59 | 17,39 |
| 12/1977 | 28,51 | 23,27 | 19,56 |
| 01/1978 | 30,17 | 24,55 | 20,39 |
| 02/1978 | 28,98 | 23,8 | 19,97 |
| 03/1978 | 28,65 | 23,36 | 19,7 |
| 04/1978 | 24,59 | 18,35 | 13,58 |
| 05/1978 | 20,88 | 14,7 | 10,43 |
| 06/1978 | 20,36 | 14,42 | 10,41 |
| 07/1978 | 20,52 | 15,83 | 12,55 |
| 08/1978 | 19,77 | 14,69 | 11,11 |
| 09/1978 | 22,77 | 17,53 | 13,81 |
| 10/1978 | 25,19 | 20,04 | 16,43 |
| 11/1978 | 25,92 | 20,78 | 16,92 |
| 12/1978 | 28,93 | 23,51 | 19,24 |
| 01/1979 | 29,35 | 23,25 | 18,7 |
| 02/1979 | 30,67 | 24,55 | 20,55 |
| 03/1979 | 26,5 | 21,73 | 18,26 |
| 04/1979 | 22,81 | 18,54 | 15,29 |
| 05/1979 | 20,19 | 15,22 | 11,5 |
| 06/1979 | 18,07 | 11,77 | 7,3 |
| 07/1979 | 19,59 | 14,33 | 10,34 |
| 08/1979 | 20,81 | 16,46 | 13,23 |
| 09/1979 | 21,32 | 15,99 | 11,47 |
| 10/1979 | 24 | 19,34 | 15,89 |
| 11/1979 | 26,08 | 20,78 | 16,25 |
| 12/1979 | 28,1 | 23,19 | 19,3 |
| 01/1980 | 29,07 | 23,59 | 18,95 |
| 02/1980 | 29,72 | 24,51 | 20,72 |
| 03/1980 | 30,03 | 25,29 | 21,92 |
| 04/1980 | 27,58 | 22,03 | 18,1 |
| 05/1980 | 23,61 | 18,58 | 14,98 |
| 08/1980 | 19,84 | 15,02 | 11,1 |
| 09/1980 | 20,9 | 14,53 | 9,73 |
| 10/1980 | 23,86 | 18,98 | 15,47 |
| 11/1980 | 26,37 | 20,84 | 16,52 |
| 12/1980 | 27,91 | 23,06 | 19,3 |
| 01/1981 | 30,07 | 24,71 | 20,67 |
| 02/1981 | 31,12 | 25,75 | 21,57 |
| 03/1981 | 27,79 | 22,44 | 18,3 |
| 04/1981 | 26,51 | 20,64 | 16,15 |
| 05/1981 | 26,17 | 20,35 | 15,7 |
| 07/1981 | 20,68 | 15,54 | 11,71 |
| 08/1981 | 22,94 | 16,53 | 11,42 |
| 09/1981 | 21,91 | 16,8 | 12,69 |
| 10/1981 | 23,66 | 18,32 | 13,45 |
| 11/1981 | 26,8 | 21,73 | 17,99 |
| 12/1981 | 28,32 | 22,46 | 17,96 |
| 01/1982 | 29,42 | 23,53 | 19 |
| 02/1982 | 29,58 | 24,47 | 20,87 |
| 03/1982 | 28,95 | 23,3 | 19,39 |
| 04/1982 | 26,54 | 20,81 | 16,46 |
| 05/1982 | 22,07 | 16,62 | 12,6 |
| 06/1982 | 21 | 15,62 | 11,29 |
| 07/1982 | 18,8 | 13,58 | 10,09 |
| 08/1982 | 20,16 | 15,41 | 11,26 |
| 09/1982 | 23,52 | 18,39 | 14,35 |
| 10/1982 | 23,57 | 18,19 | 13,83 |
| 11/1982 | 25,19 | 20,35 | 16,56 |
| 12/1982 | 28,42 | 22,99 | 18,43 |
| 01/1983 | 29,75 | 24,98 | 21,76 |
| 02/1983 | 28,99 | 24,17 | 20,74 |
| 03/1983 | 26,22 | 21,59 | 18,17 |
| 04/1983 | 24,25 | 19,83 | 16,74 |
| 05/1983 | 21,38 | 17,55 | 14,86 |
| 06/1983 | 17,35 | 12,74 | 8,9 |
| 07/1983 | 17,08 | 13,23 | 10,39 |
| 08/1983 | 20,51 | 15,54 | 11,82 |
| 09/1983 | 20,9 | 15,44 | 11,28 |
| 10/1983 | 25,3 | 19,58 | 15,12 |
| 11/1983 | 27,18 | 21,86 | 17,63 |
| 12/1983 | 30,26 | 24,3 | 20,03 |
| 01/1984 | 31,08 | 25,93 | 22,26 |
| 02/1984 | 33,31 | 26,84 | 22,6 |
| 03/1984 | 28,32 | 23,37 | 19,81 |
| 04/1984 | 24,48 | 19,54 | 15,88 |
| 05/1984 | 22,27 | 17,66 | 14,17 |
| 06/1984 | 18,44 | 14,15 | 10,92 |
| 07/1984 | 19,9 | 14,45 | 10,17 |
| 08/1984 | 18,01 | 12,98 | 9,27 |
| 09/1984 | 21,3 | 16,36 | 12,81 |
| 10/1984 | 26,06 | 19,98 | 15,63 |
| 11/1984 | 26,14 | 20,66 | 16,54 |
| 12/1984 | 27,18 | 21,3 | 16,7 |
| 01/1988 | 30,16 | 24,71 | 20,93 |
| 02/1988 | 29,54 | 23,92 | 20,09 |
| 03/1988 | 32,04 | 25,59 | 21,05 |
| 04/1988 | 25,03 | 18,93 | 14,56 |
| 05/1988 | 19,07 | 13,95 | 10,3 |
| 06/1988 | 17,69 | 11,76 | 7,16 |
| 07/1988 | 19,4 | 13 | 8,29 |
| 08/1988 | 20,56 | 15,41 | 11,62 |
| 09/1988 | 19,95 | 15,7 | 12,55 |
| 10/1988 | 24,64 | 18,39 | 13,31 |
| 11/1988 | 27 | 20,74 | 15,6 |
| 12/1988 | 30,25 | 24,1 | 19,03 |
| 01/1989 | 30,17 | 24,62 | 20,39 |
| 02/1989 | 30,88 | 24,94 | 20,36 |
| 03/1989 | 28,82 | 23,25 | 19,08 |
| 04/1989 | 25,6 | 20,49 | 16,48 |
| 05/1989 | 22,37 | 16,5 | 12,09 |
| 06/1989 | 19,72 | 14,82 | 11,15 |
| 07/1989 | 17,91 | 12,29 | 8,32 |
| 08/1989 | 22,46 | 15,85 | 10,35 |
| 09/1989 | 20,87 | 15,65 | 11,47 |
| 10/1989 | 24,29 | 18,08 | 13,01 |
| 11/1989 | 27,26 | 21,33 | 16,39 |
| 12/1989 | 30,63 | 24,26 | 19,56 |
| 01/1990 | 31 | 24,92 | 20,31 |
| 02/1990 | 29,96 | 24,41 | 20,55 |
| 03/1990 | 28,52 | 23,22 | 19,38 |
| 04/1990 | 25,53 | 21,04 | 17,62 |
| 05/1990 | 21,39 | 15,47 | 11,02 |
| 06/1990 | 18,06 | 12,4 | 8,12 |
| 07/1990 | 17,67 | 12,5 | 8,75 |
| 08/1990 | 22,3 | 15,62 | 10,26 |
| 09/1990 | 20,18 | 15,26 | 11,13 |
| 10/1990 | 26,27 | 20,86 | 16,7 |
| 11/1990 | 28,72 | 22,96 | 18,37 |
| 12/1990 | 28,57 | 22,54 | 17,61 |
| 01/1991 | 30,23 | 23,67 | 18,7 |
| 02/1991 | 31,03 | 24,02 | 18,41 |
| 03/1991 | 30,55 | 24,11 | 19,28 |
| 04/1991 | 26,15 | 20,55 | 16,69 |
| 05/1991 | 25,31 | 19,41 | 15,47 |
| 06/1991 | 20,32 | 15,22 | 11,71 |
| 07/1991 | 19,75 | 13,83 | 9,43 |
| 08/1991 | 21,81 | 16,39 | 12,69 |
| 09/1991 | 23,99 | 17,85 | 13,64 |
| 10/1991 | 26,17 | 20,37 | 15,85 |
| 11/1991 | 26,93 | 21,27 | 17,14 |
| 12/1991 | 30,6 | 24,82 | 20,57 |
| 01/1992 | 30,43 | 24,51 | 20,08 |
| 02/1992 | 31,48 | 25,79 | 21,9 |
| 03/1992 | 30,05 | 24,03 | 19,81 |
| 04/1992 | 25,52 | 20,46 | 17,01 |
| 05/1992 | 21,44 | 16,38 | 12,8 |
| 06/1992 | 22,68 | 16,41 | 12,13 |
| 07/1992 | 16,91 | 11,87 | 8,08 |
| 08/1992 | 20,5 | 14,3 | 9,75 |
| 09/1992 | 22,14 | 16,74 | 12,77 |
| 10/1992 | 26,16 | 19,53 | 13,94 |
| 11/1992 | 27,16 | 20,63 | 15,42 |
| 12/1992 | 29,95 | 23,13 | 18,06 |
| 01/1993 | 31,34 | 25,06 | 20,38 |
| 02/1993 | 29,28 | 23,6 | 19,3 |
| 03/1993 | 28,22 | 22,88 | 19,01 |
| 04/1993 | 27,66 | 21,73 | 17,2 |
| 05/1993 | 23,29 | 17,15 | 12,84 |
| 06/1993 | 19,13 | 13,55 | 9,3 |
| 07/1993 | 17,97 | 12,93 | 8,95 |
| 08/1993 | 21,9 | 14,36 | 8,52 |
| 09/1993 | 20,83 | 15,81 | 11,63 |
| 10/1993 | 26,4 | 20,36 | 15,62 |
| 11/1993 | 28,07 | 22,16 | 17,28 |
| 12/1993 | 29,73 | 23,37 | 18,44 |
| 01/1994 | 30,33 | 23,91 | 18,75 |
| 02/1994 | 28,85 | 23,65 | 20,17 |
| 03/1994 | 28,05 | 22,51 | 18,05 |
| 04/1994 | 25,51 | 19,21 | 14,6 |
| 05/1994 | 24,33 | 19,13 | 15,66 |
| 06/1994 | 20,09 | 14,33 | 10,22 |
| 07/1994 | 20,41 | 15,07 | 11,42 |
| 08/1994 | 21,28 | 15,19 | 11,02 |
| 09/1994 | 25,21 | 18,43 | 13,62 |
| 10/1994 | 24,6 | 19,52 | 15,77 |
| 11/1994 | 26,53 | 21,01 | 16,86 |
| 12/1994 | 32,05 | 25,2 | 20,12 |
| 01/1995 | 30,74 | 25,04 | 21,16 |
| 02/1995 | 29,46 | 23,65 | 19,72 |
| 03/1995 | 29,12 | 23,04 | 18,57 |
| 04/1995 | 26,22 | 19,77 | 15,23 |
| 05/1995 | 21,95 | 16,14 | 12,4 |
| 06/1995 | 20,45 | 14,29 | 10,27 |
| 07/1995 | 21,6 | 16,12 | 12,48 |
| 08/1995 | 21,55 | 15,72 | 12,03 |
| 09/1995 | 22,62 | 16,79 | 12,53 |
| 10/1995 | 24,17 | 18,44 | 14,03 |
| 11/1995 | 28,93 | 22,66 | 17,99 |
| 12/1995 | 30,44 | 24,12 | 19,37 |
| 01/1996 | 29,89 | 24,29 | 20,51 |
| 02/1996 | 29,78 | 24,18 | 20,53 |
| 03/1996 | 28,67 | 23,09 | 19,58 |
| 04/1996 | 27,03 | 21,03 | 17,24 |
| 05/1996 | 22,94 | 17,01 | 13,03 |
| 06/1996 | 17,76 | 12,3 | 8,69 |
| 07/1996 | 16,99 | 11,24 | 7,39 |
| 08/1996 | 22,59 | 15,55 | 11,07 |
| 09/1996 | 21,4 | 15,93 | 12,01 |
| 10/1996 | 25,34 | 19,88 | 16,12 |
| 11/1996 | 28,4 | 22,38 | 18,32 |
| 12/1996 | 29,87 | 24,07 | 20,14 |
| 01/1997 | 32,09 | 25,76 | 21,69 |
| 02/1997 | 29,62 | 24,4 | 21,11 |
| 03/1997 | 28,63 | 22,43 | 18,03 |
| 04/1997 | 26,56 | 20,06 | 15,75 |
| 05/1997 | 22,95 | 16,8 | 12,79 |
| 06/1997 | 19,42 | 14,19 | 10,43 |
| 07/1997 | 20,67 | 15,3 | 11,55 |
| 08/1997 | 23,06 | 16,93 | 12,82 |
| 09/1997 | 22,32 | 17,16 | 13,52 |
| 10/1997 | 23,27 | 18,95 | 16,01 |
| 11/1997 | 26,51 | 21,26 | 17,78 |
| 12/1997 | 30,28 | 24,47 | 20,47 |
| 01/1998 | 29,13 | 24,07 | 20,7 |
| 02/1998 | 28,26 | 23,66 | 20,75 |
| 03/1998 | 27,2 | 22,21 | 18,49 |
| 04/1998 | 24,03 | 19,87 | 17,28 |
| 05/1998 | 22 | 16,54 | 13,3 |
| 06/1998 | 19,6 | 13,93 | 10,1 |
| 07/1998 | 20,47 | 15,31 | 12,25 |
| 08/1998 | 19,77 | 15,81 | 13,19 |
| 09/1998 | 21,22 | 16,43 | 13,1 |
| 10/1998 | 25,19 | 19,43 | 15,32 |
| 11/1998 | 28,24 | 21,43 | 16,33 |
| 12/1998 | 28,91 | 22,82 | 18,35 |
| 01/1999 | 30,65 | 24,63 | 20,53 |
| 02/1999 | 29,56 | 23,82 | 19,94 |
| 03/1999 | 31,64 | 25,02 | 20,94 |
| 04/1999 | 24 | 18,86 | 15,6 |
| 05/1999 | 21,51 | 16,06 | 12,29 |
| 06/1999 | 18,87 | 13,62 | 10,38 |
| 07/1999 | 18,78 | 13,94 | 10,86 |
| 08/1999 | 21,94 | 15,4 | 10,8 |
| 09/1999 | 22,65 | 17,05 | 12,93 |
| 10/1999 | 23,07 | 18,19 | 14,69 |
| 11/1999 | 26,36 | 20,31 | 15,76 |
| 12/1999 | 29,08 | 23,07 | 18,88 |
| 01/2000 | 30,63 | 24,83 | 20,35 |
| 02/2000 | 29,93 | 24,04 | 19,94 |
| 03/2000 | 27,85 | 22,4 | 18,49 |
| 04/2000 | 26,32 | 20,85 | 17 |
| 05/2000 | 21,78 | 16,59 | 13,2 |
| 06/2000 | 21,5 | 16,24 | 12,92 |
| 07/2000 | 17,59 | 11,32 | 7,25 |
| 08/2000 | 20,85 | 14,04 | 9,14 |
| 09/2000 | 22,25 | 16,56 | 12,35 |
| 10/2000 | 24,02 | 19,4 | 16,14 |
| 11/2000 | 26,87 | 21,06 | 16,75 |
| 12/2000 | 29,51 | 23,64 | 19,12 |
| 10/2001 | 26,62 | 21,35 | 17,48 |
| 11/2001 | 27,92 | 21,95 | 17,52 |
| 12/2001 | 28,73 | 22,84 | 18,9 |
| 01/2002 | 30,44 | 25,03 | 20,94 |
| 02/2002 | 30,14 | 23,65 | 19,7 |
| 03/2002 | 31,27 | 25,91 | 22,12 |
| 04/2002 | 25,49 | 21,15 | 17,6 |
| 05/2002 | 23,81 | 18,92 | 15,97 |
| 06/2002 | 19,79 | 14,86 | 11,63 |
| 07/2002 | 19,06 | 14,05 | 11,1 |
| 08/2002 | 21,91 | 16,83 | 13,29 |
| 09/2002 | 21,17 | 15,72 | 11,57 |
| 10/2002 | 25,44 | 20,88 | 17,47 |
| 11/2002 | 27,36 | 22,21 | 18,29 |
| 12/2002 | 29,04 | 23,65 | 19,79 |
| 01/2003 | 31,03 | 25,08 | 20,72 |
| 02/2003 | 31,33 | 25,62 | 22 |
| 03/2003 | 29,69 | 23,89 | 20,37 |
| 04/2003 | 25,23 | 19,89 | 16,26 |
| 05/2003 | 23,15 | 17,29 | 13,76 |
| 06/2003 | 20,82 | 16,5 | 14,05 |
| 07/2003 | 20,05 | 14,63 | 11,42 |
| 08/2003 | 20,41 | 14,15 | 9,89 |
| 09/2003 | 22,52 | 16,24 | 12,15 |
| 10/2003 | 26,04 | 20,01 | 15,83 |
| 11/2003 | 27,46 | 21,55 | 17,31 |
| 12/2003 | 27,85 | 22,4 | 18,46 |
| 01/2004 | 30,83 | 24,68 | 20,5 |
| 02/2004 | 29,34 | 23,69 | 19,61 |
| 03/2004 | 28,93 | 22,92 | 18,84 |
| 04/2004 | 28,47 | 22,08 | 18,18 |
| 05/2004 | 20,65 | 15,93 | 13,07 |
| 06/2004 | 21,61 | 15,86 | 12,39 |
| 07/2004 | 19,49 | 13,54 | 9,71 |
| 08/2004 | 21,37 | 15,36 | 11,13 |
| 09/2004 | 23,99 | 18,55 | 14,83 |
| 10/2004 | 25,06 | 18,8 | 14,06 |
| 11/2004 | 26,63 | 21,07 | 16,98 |
| 12/2004 | 29,17 | 23,08 | 18,77 |
| 01/2005 | 32,22 | 25,44 | 20,65 |
| 02/2005 | 30,5 | 24,51 | 20,82 |
| 03/2005 | 29,82 | 23,82 | 19,8 |
| 04/2005 | 25,37 | 20,1 | 16,64 |
| 05/2005 | 23,37 | 18,14 | 14,62 |
| 06/2005 | 23,14 | 18,18 | 15,11 |
| 07/2005 | 20,88 | 14,91 | 10,83 |
| 08/2005 | 22,96 | 16,88 | 13,39 |
| 09/2005 | 20,23 | 15,7 | 12,42 |
| 10/2005 | 24,07 | 19,13 | 15,44 |
| 11/2005 | 28,33 | 22,06 | 17,15 |
| 12/2005 | 28,94 | 22,95 | 18,4 |
| 01/2006 | 31,26 | 25,55 | 21,6 |
| 02/2006 | 29,79 | 24,44 | 20,76 |
| 03/2006 | 29,63 | 23,93 | 19,96 |
| 04/2006 | 25,86 | 20,19 | 16,47 |
| 05/2006 | 20,66 | 15,45 | 12,15 |
| 06/2006 | 21,49 | 15,62 | 12,21 |
| 07/2006 | 22,5 | 17,04 | 13,68 |
| 08/2006 | 20,98 | 15,04 | 11,01 |
| 09/2006 | 22,13 | 16,09 | 11,91 |
| 10/2006 | 27,33 | 20,64 | 16,21 |
| 11/2006 | 26,74 | 20,93 | 17,11 |
| 12/2006 | 31,58 | 25,13 | 20,57 |
| 01/2007 | 31,08 | 25,32 | 21,26 |
| 02/2007 | 30,81 | 24,94 | 20,5 |
| 03/2007 | 30,28 | 24,99 | 21,59 |
| 04/2007 | 27,79 | 22,02 | 18,36 |
| 05/2007 | 20,21 | 15,14 | 11,95 |
| 06/2007 | 18,81 | 14,27 | 11,35 |
| 07/2007 | 17,79 | 12,16 | 8,49 |
| 08/2007 | 19,99 | 14,26 | 10,29 |
| 09/2007 | 24,97 | 19,16 | 15,47 |
| 10/2007 | 26,03 | 20,87 | 17,18 |
| 11/2007 | 26,55 | 20,34 | 15,25 |
| 12/2007 | 30,55 | 24,03 | 19,14 |
| 01/2008 | 30,4 | 24,46 | 20,05 |
| 02/2008 | 29,76 | 23,97 | 20,02 |
| 03/2008 | 29,7 | 23,83 | 19,74 |
| 04/2008 | 25,6 | 19,65 | 15,61 |
| 05/2008 | 22,35 | 16,65 | 12,82 |
| 06/2008 | 18,09 | 13,01 | 9,82 |
| 07/2008 | 21,22 | 16,22 | 13,06 |
| 08/2008 | 21,09 | 15,47 | 11,44 |
| 09/2008 | 21,33 | 15,96 | 12,06 |
| 10/2008 | 24,14 | 19,25 | 15,76 |
| 11/2008 | 27,83 | 21,86 | 17,69 |
| 12/2008 | 29,14 | 23,12 | 18,45 |
| 01/2009 | 29,05 | 23,55 | 19,43 |
| 02/2009 | 30,23 | 24,72 | 20,69 |
| 03/2009 | 29,25 | 23,67 | 20,02 |
| 04/2009 | 27,55 | 20,84 | 16,23 |
| 05/2009 | 23,89 | 17,75 | 13,71 |
| 06/2009 | 18,86 | 13,28 | 9,65 |
| 07/2009 | 17,5 | 11,96 | 8,04 |
| 08/2009 | 23,14 | 16,69 | 11,83 |
| 09/2009 | 21,99 | 17,05 | 13,81 |
| 10/2009 | 25,13 | 18,92 | 14,47 |
| 11/2009 | 27,97 | 23,22 | 19,76 |
| 12/2009 | 28,95 | 23,76 | 19,94 |
| 01/2010 | 30,28 | 25,14 | 21,25 |
| 02/2010 | 32,41 | 26,44 | 22,23 |
| 03/2010 | 28,97 | 23,69 | 19,75 |
| 04/2010 | 25,98 | 20,16 | 15,68 |
| 05/2010 | 21,45 | 17,28 | 14,32 |
| 06/2010 | 20,46 | 15,06 | 11,14 |
| 07/2010 | 20,75 | 14,45 | 10,02 |
| 08/2010 | 20 | 14,63 | 10,95 |
| 09/2010 | 22 | 17,52 | 14,28 |
| 10/2010 | 23,93 | 18,41 | 14,02 |
| 11/2010 | 27,52 | 21,01 | 16,13 |
| 12/2010 | 29,74 | 23,57 | 18,98 |
| 01/2011 | 32,02 | 26,17 | 22,56 |
| 02/2011 | 30,19 | 25,07 | 21,73 |
| 03/2011 | 28,84 | 22,98 | 19,17 |
| 04/2011 | 26,69 | 20,45 | 16,53 |
| 05/2011 | 22,23 | 16,6 | 13,22 |
| 06/2011 | 18,82 | 13,68 | 10,41 |
| 07/2011 | 18,36 | 13,18 | 9,98 |
| 08/2011 | 19,95 | 14,75 | 11,41 |
| 09/2011 | 23,51 | 16,71 | 11,97 |
| 10/2011 | 25,78 | 19,85 | 15,8 |
| 11/2011 | 28,84 | 21,91 | 17,05 |
| 12/2011 | 29,63 | 22,88 | 18,4 |
| 01/2012 | 31,25 | 24,76 | 20,16 |
| 02/2012 | 33,57 | 26,6 | 22,3 |
| 03/2012 | 30,34 | 23,55 | 18,78 |
| 04/2012 | 26,11 | 19,86 | 16,02 |
| 05/2012 | 25,46 | 18,59 | 14,43 |
| 06/2012 | 21,25 | 14,76 | 10,55 |
| 07/2012 | 19,71 | 13,31 | 9,2 |
| 08/2012 | 26,25 | 19,1 | 14,56 |
| 09/2012 | 24,04 | 18,2 | 14,16 |
| 10/2012 | 27,07 | 21,25 | 17,35 |
| 11/2012 | 30,29 | 23,3 | 18,1 |
| 12/2012 | 32,77 | 25,67 | 20,14 |
| 01/2013 | 31,42 | 24,2 | 19,06 |
| 02/2013 | 30,31 | 24,58 | 20,81 |
| 03/2013 | 26,85 | 21,73 | 18,24 |
| 04/2013 | 26,46 | 20,28 | 16,07 |
| 05/2013 | 22,24 | 16,44 | 12,75 |
| 06/2013 | 19,46 | 14,56 | 11,49 |
| 07/2013 | 19,85 | 13,63 | 9,42 |
| 08/2013 | 19,45 | 13,7 | 9,76 |
| 09/2013 | 23,23 | 17,52 | 13,64 |
| 10/2013 | 24,94 | 19,22 | 15,01 |
| 11/2013 | 28,59 | 22,3 | 17,47 |
| 12/2013 | 31,34 | 24,92 | 20,27 |
| 01/2014 | 33,09 | 26,62 | 21,93 |
| 02/2014 | 32,35 | 26,17 | 21,85 |
| 03/2014 | 28,28 | 23,08 | 19,41 |
| 04/2014 | 26,48 | 21,08 | 17,41 |
| 05/2014 | 21,68 | 16,76 | 13,67 |
| 06/2014 | 19,87 | 15,25 | 12,11 |
| 07/2014 | 21,17 | 15,33 | 11,29 |
| 08/2014 | 22,8 | 15,93 | 11,51 |
| 09/2014 | 24 | 18,62 | 14,88 |
| 10/2014 | 26,73 | 21,13 | 16,97 |
| 11/2014 | 28,88 | 22,93 | 18,42 |
| 12/2014 | 29,59 | 24,38 | 20,36 |
| 01/2015 | 30,89 | 25,63 | 21,66 |
| 02/2015 | 30,23 | 24,96 | 21,17 |
| 03/2015 | 29,52 | 24 | 20,13 |
| 04/2015 | 26,8 | 21,05 | 17,08 |
| 05/2015 | 23,61 | 18,29 | 14,75 |
| 06/2015 | 21,18 | 15,46 | 11,59 |
| 07/2015 | 20,2 | 15,67 | 12,68 |
| 08/2015 | 26,17 | 20,19 | 16,22 |
| 09/2015 | 22,08 | 17,29 | 13,83 |
| 10/2015 | 23,61 | 18,86 | 15,4 |
| 11/2015 | 25,7 | 20,96 | 17,52 |
| 12/2015 | 29,01 | 23,96 | 20,2 |
| 01/2016 | 32,02 | 25,85 | 21,33 |
| 02/2016 | 31,81 | 25,97 | 21,81 |
| 03/2016 | 27,83 | 22,9 | 19,49 |
| 04/2016 | 27,12 | 22,79 | 19,25 |
| 05/2016 | 19,61 | 15,25 | 12,25 |
| 06/2016 | 16,41 | 11,55 | 8,32 |
| 07/2016 | 20,08 | 14,2 | 10,42 |
| 08/2016 | 22,04 | 16,21 | 12,07 |
| 09/2016 | 22,06 | 16,38 | 12,4 |
| 10/2016 | 24,85 | 19,38 | 15,45 |
| 11/2016 | 27,54 | 21,17 | 15,86 |
| 12/2016 | 30,95 | 24,7 | 19,97 |
